# ESCRT-III mediates budding across the inner nuclear membrane and regulates its integrity

**DOI:** 10.1038/s41467-018-05889-9

**Published:** 2018-08-23

**Authors:** Jun Arii, Mizuki Watanabe, Fumio Maeda, Noriko Tokai-Nishizumi, Takahiro Chihara, Masayuki Miura, Yuhei Maruzuru, Naoto Koyanagi, Akihisa Kato, Yasushi Kawaguchi

**Affiliations:** 1 Division of Molecular Virology, Department of Microbiology and Immunology, The Institute of Medical Science, The University of Tokyo, Minato-ku, Tokyo, 108-8639 Japan; 2 Department of Infectious Disease Control, International Research Center for Infectious Diseases, The Institute of Medical Science, The University of Tokyo, Minato-ku, Tokyo, 108-8639 Japan; 3 Microscope Core Laboratory, The Institute of Medical Science, The University of Tokyo, Minato-ku, Tokyo, 108-8639 Japan; 4 Department of Genetics, Graduate School of Pharmaceutical Sciences, The University of Tokyo, Tokyo, 113-0033 Japan; 5 Present Address: Graduate School of Science, Hiroshima University, Higashi-Hiroshima, Hiroshima, 739-8526 Japan

## Abstract

Vesicle-mediated nucleocytoplasmic transport is a nuclear pore-independent mechanism for the nuclear export of macromolecular complexes, but the molecular basis for this transport remains largely unknown. Here we show that endosomal sorting complex required for transport-III (ESCRT-III) is recruited to the inner nuclear membrane (INM) during the nuclear export of herpes simplex virus 1 (HSV-1). Scission during HSV-1 budding through the INM is prevented by depletion of ESCRT-III proteins. Interestingly, in uninfected human cells, the depletion of ESCRT-III proteins induces aberrant INM proliferation. Our results show that HSV-1 expropriates the ESCRT-III machinery in infected cells for scission of the INM to produce vesicles containing progeny virus nucleocapsids. In uninfected cells, ESCRT-III regulates INM integrity by downregulating excess INM.

## Abstract (web summary)

The endosomal sorting complex required for transport-III (ESCRT-III) has been implicated in the packaging of HIV and HSV-1 viruses in the cytoplasm. Here the authors show that ESCRT-III proteins are required for the transport of HSV-1 nucleocapsids from nucleoplasm to cytosol through the nuclear envelope and confirm that the same mechanism is also used for the nucleocytoplasmic transport of RNP in Drosophila cells.

## Introduction

Vesicle-mediated nucleocytoplasmic transport is a unique mechanism for the nuclear export of macromolecular complexes, in which a macromolecular complex in the nucleus buds through the inner nuclear membrane (INM) to form a vesicle in the perinuclear space (primary envelopment), which then fuses with the outer nuclear membrane (ONM) to release the complex into the cytoplasm (de-envelopment)^1^. This type of transport is observed in herpesvirus-infected mammalian cells for the nuclear export of viral nucleocapsids^2^, but is not common in other types of cells. However, *Drosophila* cellular large ribonucleoprotein complexes (RNPs) have been reported to utilize this nuclear export mechanism^3^, indicating that it can be carried out solely by intrinsic cellular machinery, and that herpesviruses may expropriate this transport mechanism. Although several cellular regulatory proteins (e.g., protein kinase C enzymes, CD98 heavy chain, β1 integrin, and p32) involved in disintegration of the nuclear lamina to facilitate herpesvirus nucleocapsid access to the INM and in herpesvirus de-envelopment have been implicated^4–6^, the molecular mechanism(s) for vesicle-mediated nucleocytoplasmic transport remains largely unknown. In particular, there is a lack of information regarding cellular proteins that directly regulate primary envelopment, in which the INM is deformed to wrap around a macromolecular complex followed by scission of the INM to complete vesicle formation.

Endosomal sorting complex required for transport-III (ESCRT-III) functions in a number of cellular processes, including extracellular microvesicle formation, enveloped virus budding, and the abscission stage of cytokinesis^7^. In each case, abscission (e.g., of endosomal and plasma membranes, and of the midbody) is thought to be caused by the polymerization of ESCRT-III components to remodel the membrane^7^. Monomeric ESCRT-III proteins are usually soluble but once their auto-inhibition is relieved they assemble into membrane-bound filaments that have critical roles in membrane fission^7^. ESCRT-II, ALIX, and charged multivesicular body protein (CHMP) 7 have been identified as upstream factors of ESCRT-III that recruit ESCRT-III proteins and initiate their assembly^8^. ALIX and/or ESCRT-I are also known to bind regulators in various pathway-specific signals mediated by the ESCRT-III machinery: these interactions are required to recruit ESCRT-III proteins to their sites of action^8^. These regulators include ubiquitin for multivesicular body (MVB) formation, CEP55 for cytokinesis, and GAG p6 for human immunodeficiency virus 1 (HIV-1) budding^8^. VPS4 AAA-ATPases disassemble ESCRT-III filaments to the monomeric state, which is essential in recycling ESCRT-III proteins for further rounds of assembly.

Recent reports have identified novel ESCRT-III functions in the nucleus including resealing nuclear membranes (NMs) during late anaphase^9,10^, quality control of nuclear pore complex (NPC) assembly^11,12^, and repair of NM ruptures produced during migration of cancer and immune cells^13,14^. Data have accumulated, suggesting that the ESCRT-II/ESCRT-III hybrid protein CHMP7, but not ESCRT-I or ALIX, is required for NM reformation by recruiting ESCRT-III machinery to the NM^9,14^. However, the mechanism by which ESCRT-III acts in the nucleus, including whether ESCRT-III proteins remodel NMs during these processes, is unknown.

Scission of the INM can be easily observed during the nuclear export of herpesvirus nucleocapsids (nuclear egress)^2^. Therefore, we investigated whether ESCRT-III mediates herpes simplex virus 1 (HSV-1) nuclear transport, a process typical of herpesviruses that produce life-long infections in humans, causing various mucocutaneous diseases and encephalitis^15^. Here we show that ESCRT-III promotes HSV-1 primary envelopment by mediating scission during HSV-1 budding through the INM. We also present data showing ESCRT-III downregulates INM proliferation in uninfected human cells. These results identify functions of ESCRT-III in the vesicle-mediated nucleocytoplasmic transport of HSV-1 and in the regulation of INM integrity in uninfected cells.

## Results

### HSV-1 infection recruits ESCRT-III to the INM

**Fig. 1:**
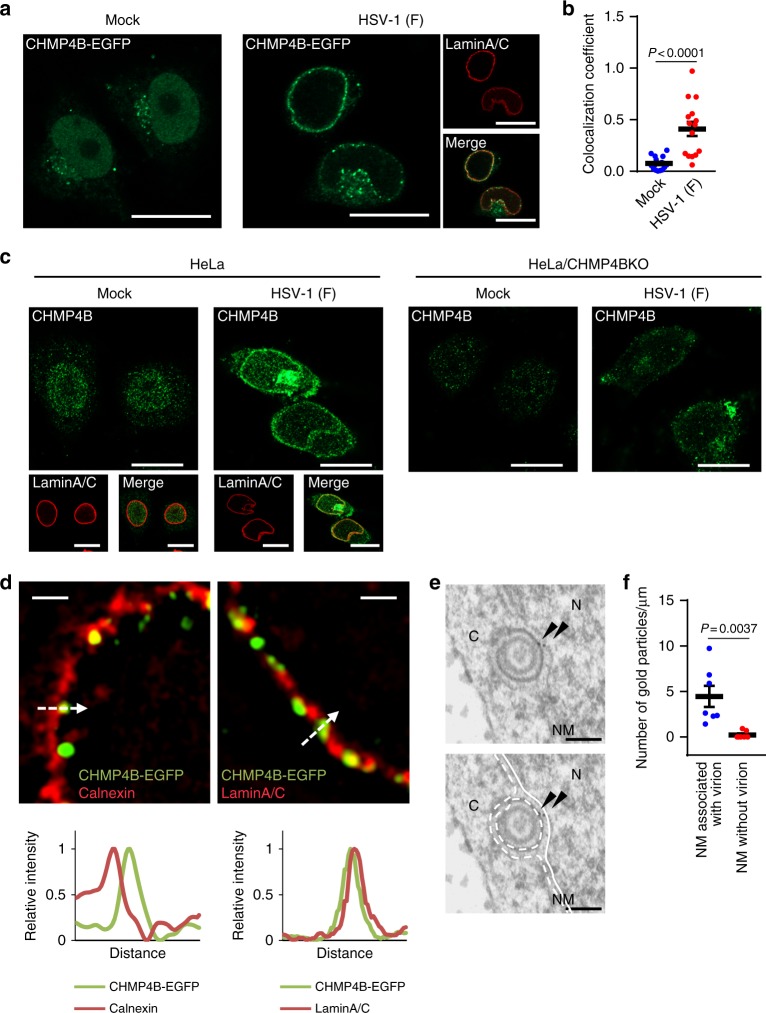
ESCRT-III is recruited to the nuclear rim in HSV-1-infected cells. **a** HeLa/CHMP4B-EGFP cells mock-infected or infected with HSV-1 for 22 h were analyzed by confocal microscopy for CHMP4B-EGFP and lamin A/C. Bars, 20 μm. Images are representative of three independent experiments. **b** Colocalization between CHMP4B-EGFP and lamin A/C in the experiment in (**a**) was quantified using Mander’s colocalization coefficient. Data are shown as the mean ± SEM (*n* = 16 for mock-infected and 15 for HSV-1-infected cells representative of three independent experiments). **c** HeLa or HeLa/CHMP4B KO cells were mock-infected or infected with HSV-1 for 22 h and analyzed by confocal microscopy for CHMP4B and lamin A/C. Bars, 20 μm. Images are representative of three independent experiments. **d** HeLa/CHMP4B-EGFP cells infected with HSV-1 for 22 h were analyzed by N-SIM super-resolution microscopy for (left) CHMP4B-EGFP and calnexin or (right) CHMP4B-EGFP and lamin A/C. Bars, 1 μm. Fluorescence line scans along the dotted lines of N-SIM images are shown under each image. Images are representative of three independent experiments. **e** HeLa/CHMP4B-EGFP cells were infected with HSV-1 for 24 h and analyzed by immunoelectron microscopy. C cytoplasm, N nucleus, NM, nuclear membrane. Bars, 200 nm. Arrowheads indicate localization of CHMP4P-EGFP labeled with anti-GFP antibody along the INM and in primary enveloped virions. In the lower panel, straight lines indicate the INM and dotted lines indicate the ONM and the envelope of virions in the perinuclear space. Images are representative of three independent experiments. **f** Quantification of gold particles on the NM with or without virions in the experiment in (**e**). Seven areas of each section were analyzed and the data are shown as the mean ± SEM. Data are representative of two independent experiments. The indicated *P*-values were obtained using the unpaired Student’s *t*-test (**b**, **f**)

To study the involvement of ESCRT-III in HSV-1 nuclear egress, we first investigated the effect of HSV-1 infection on the localization of an ESCRT-III protein, CHMP4B, using HeLa cells stably expressing CHMP4B fused to enhanced green fluorescent protein (EGFP) (Supplementary Fig. 1a, b). CHMP4 proteins are thought to be critical components of the ESCRT-III complex for membrane remodeling^16^. In agreement with a previous report^17^, the ectopic expression of CHMP4B-EGFP had no effect on the frequency of multinucleated cells and cell proliferation (Supplementary Fig. 1c, d). Moreover, CHMP4B-EGFP was localized to the midbody-like structure during cytokinesis (Supplementary Fig. 1e). These results suggest CHMP4B-EGFP is functional. In mock-infected cells, CHMP4B-EGFP was distributed in both the cytoplasm and nucleus (Fig. 1a) as previously reported^17^. In contrast, in HSV-1-infected cells, CHMP4B-EGFP redistributed to punctate structures at the nuclear rim and also colocalized with nuclear lamina protein lamin A/C (Fig. 1a, b). However, HSV-1 infection had no obvious effect on the localization of EGFP-CHMP7 (Supplementary Fig. 1f). In agreement with the localization of CHMP4B-EGFP in HSV-1-infected cells described above, endogenous CHMP4B detected by immunofluorescence was also recruited to the nuclear rim and colocalized with lamin A/C in HSV-1-infected HeLa cells (Fig. 1c), but the recruitment of fluorescence to the nuclear rim detected by CHMP4B antibody was not observed in CHMP4 knockout (KO) cells (Fig. 1c and Supplementary Fig. 1g, h). Super-resolution imaging and line plot measurements showed that punctate structures with CHMP4B-EGFP were associated with lamin A/C, but not with the endoplasmic reticulum (ER) integral protein calnexin, which is localized at the ONM and ER^18^ (Fig. 1d). In agreement with these results, immunoelectron microscopy detected CHMP4B-EGFP on the INM and primary enveloped virions in the perinuclear space (Fig. 1e). Quantification of the frequencies of CHMP4B-EGFP at the NM associated with or without virions showed that CHMP4-EGFP was significantly accumulated at the virion-associated NM (Fig. 1f). Collectively, these results indicate that HSV-1 infection specifically induced the recruitment of CHMP4B to the INM.

**Fig. 2:**
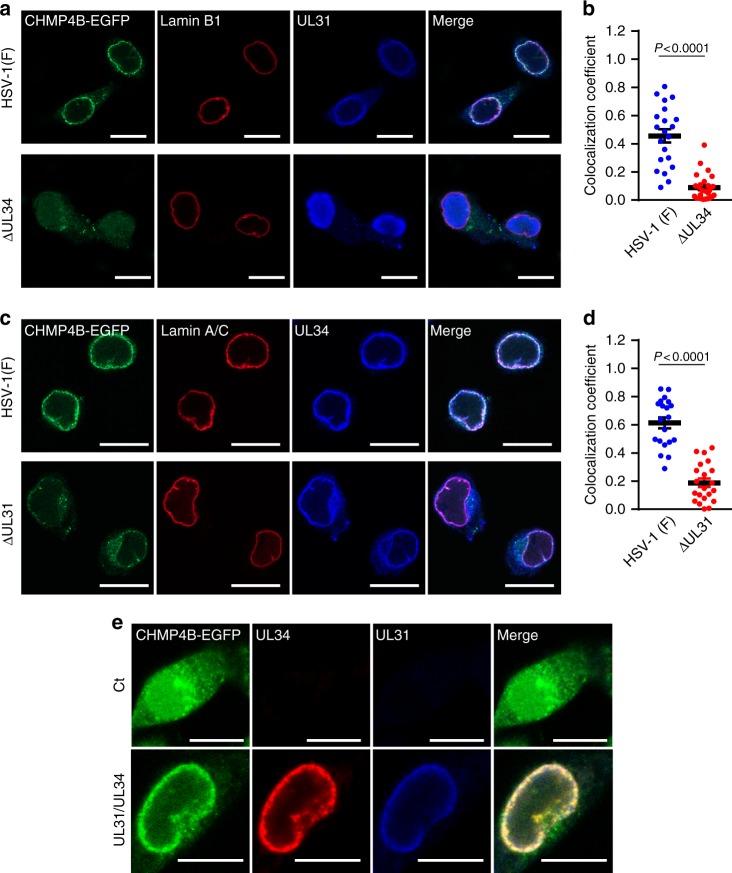
HSV-1 recruits ESCRT-III via the viral NEC. **a** Confocal microscope images of HeLa/CHMP4B-EGFP cells infected with wild-type HSV-1 or HSV-1 ΔUL34. Bars, 20 μm. Images are representative of three independent experiments. **b** Colocalization between CHMP4B-EGFP and lamin A/C in the experiment in (**a**) was quantified using Mander’s colocalization coefficient. Data are shown as the mean ± SEM (*n* = 21 for wild-type HSV-1 or 26 for HSV-1 ΔUL34-infected cells and are representative of three independent experiments). **c** Confocal microscope images of HeLa/CHMP4B-EGFP cells infected with wild-type HSV-1 or HSV-1 ΔUL31. Bars, 20 μm. Images are representative of three independent experiments. **d** Colocalization between CHMP4B-EGFP and lamin A/C was quantified using Mander’s colocalization coefficient in the experiment in (**c**). Data are shown as the mean ± SEM (*n* = 20 for wild-type HSV-1 or 21 for HSV-1 ΔUL31-infected cells in representative of three independent experiments). **e** Confocal microscope images of HeLa/CHMP4B-EGFP cells transfected with the UL31 and UL34 expression vectors. Bars, 10 μm. Images are representative of three independent experiments. The indicated *P*-values were obtained using the unpaired Student’s *t*-test (**b**, **d**)

To investigate the mechanism by which CHMP4B was recruited to the INM in HSV-1-infected cells, we studied the heterodimeric HSV-1 nuclear egress complex (NEC). NEC consists of the HSV-1 UL31 and UL34 proteins, and has a critical role in HSV-1 nuclear egress^19^. As shown in Fig. 2a, c and Supplementary Fig. 2a, b, the NEC proteins colocalized with CHMP4B-EGFP punctate structures at the nuclear rim of HSV-1-infected cells. In contrast, deletion of either NEC protein produced aberrant localization of the other NEC protein, as reported previously^20^, and impaired recruitment of CHMP4B-EGFP to the nuclear rim, i.e., colocalization efficacy between CHMP4B-EGFP, and lamin B1 or A/C was significantly reduced (Fig. 2a–d). In agreement with these results, the NEC proteins colocalized with endogenous CHMP4 at the nuclear rim of HSV-1-infected cells, and in cells infected with either the UL31-null or UL34-null mutant virus, recruitment of endogenous CHMP4B to the NM was significantly impaired (Supplementary Fig. 3). However, the ectopic expression of both UL31 and UL34 recruited CHMP4B-EGFP to the nuclear rim to colocalize with these viral proteins (Fig. 2e; Supplementary Fig. 2c). These results indicate that the HSV-1 NEC was necessary and sufficient for the recruitment of ESCRT-III to the INM.

### ESCRT-III regulates HSV-1 nuclear egress

**Fig. 3:**
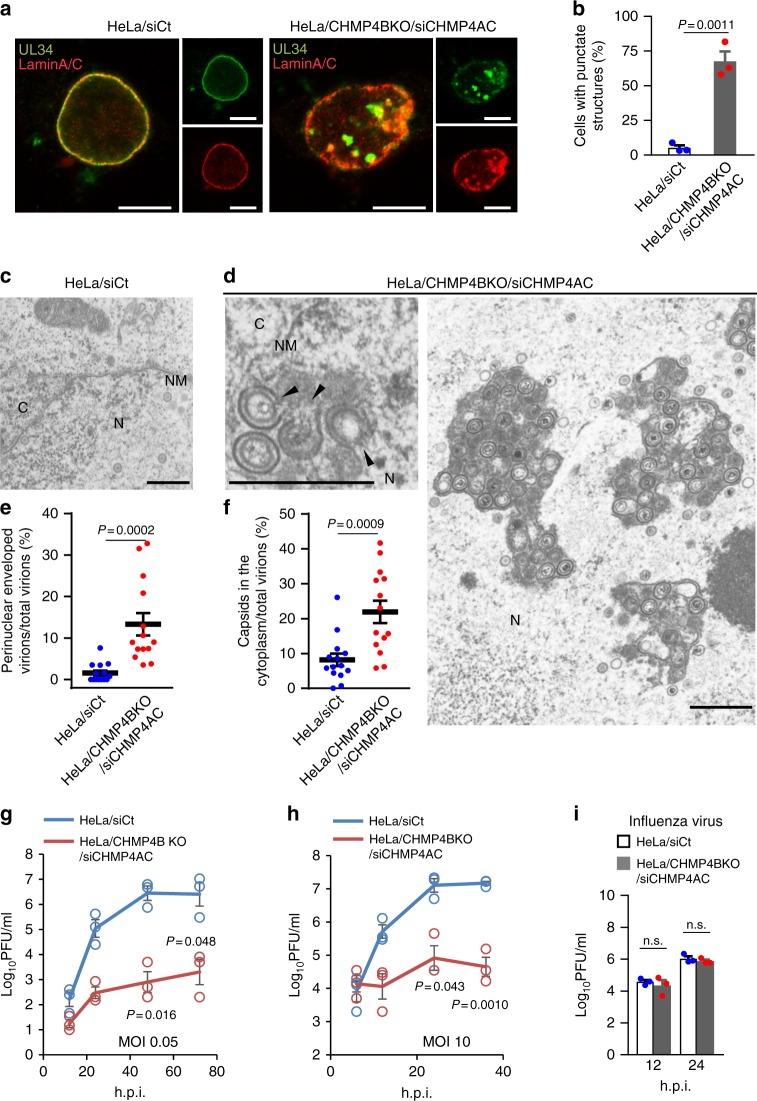
ESCRT-III is required for proper nuclear egress of HSV-1. **a** Confocal microscope images of HeLa and HeLa/CHMP4B KO cells treated with (left) control siRNA (siCt) or (Right) siRNAs to CHMP4A and CHMP4C (siCHMP4AC), respectively, for 48 h, and subsequently infected with HSV-1 for 22 h. Bars, 20 μm. Images are representative of three independent experiments. **b** Percent of cells (80–200 cells in each experiment) with aberrant punctate structures along with the nuclear rim in the experiment in (**a**). Data are shown as the mean ± SEM of three independent experiments. Electron microscope images of (**c**) HeLa and **d** HeLa/CHMP4B KO cells treated with siCt or siCHMP4AC, respectively, for 48 h and infected with HSV-1 for 22 h. Arrowheads indicate virions defective in the scission steps. C cytoplasm, N nucleus, NM nuclear membrane. Bar, 500 nm. Images are representative of three independent experiments. Percent of (**e**) perinuclear enveloped virions and **f** capsids in the cytoplasm of 14 cells in the experiments in (**c**, **d**). Data are shown as the mean ± SEM and are representative of three independent experiments. **g**–**i** HeLa and HeLa/CHMP4B KO cells treated with siRNA(s) as described in **a** were infected with HSV-1 at an MOI of (**g**) 10 or **h** 0.05, or with **i** influenza virus at an MOI of 0.01, and progeny virus titers were assayed at the indicated hours post infection (h.p.i). Data are shown as the mean ± SEM of three independent experiments. The indicated *P*-values were obtained using the unpaired Student’s *t*-test (**b**, **e**–**i**). n.s., not significant

To directly examine the role of ESCRT-III proteins in HSV-1 nuclear egress, we investigated the effects of CHMP4B KO in combination with CHMP4A and CHMP4C knockdown (KD) (Supplementary Fig. 4a, c) on the localization of UL34 and lamin A/C, and on viral morphogenesis and replication in HSV-1-infected cells. Depletion of CHMP4 proteins tended to reduce cell viability, but this was not statistically significant (Supplementary Fig. 4b). The results of depletion of CHMP4 proteins were as follows: (i) aberrant punctate structures induced at the nuclear rim and in the nucleus where UL34 and lamin A/C colocalized (Fig. 3a, b), as detected by confocal microscopy in a majority of HSV-1-infected cells; (ii) membranous structures induced that were invaginations of the INM into the nucleoplasm adjacent to the nuclear rim and in the nucleus, and which contained primary enveloped virions, as detected by electron microscopy (EM) (Fig. 3c, d; Supplementary Fig. 4d); (iii) significant accumulation of enveloped virions in the nucleus (Fig. 3e; Supplementary Table 1) and of viral capsids in the cytoplasm (Fig. 3f; Supplementary Table 1); and (iv) significant reduction of viral replication (Fig. 3g, h). In contrast, depletion of CHMP4 proteins had no effect on the replication of influenza virus (Fig. 3i). The induction of punctate structures at the NM and in the nucleus, the accumulation of enveloped virions in the nucleus and the reduction of HSV-1 replication observed by depletion of CHMP4 proteins were significantly restored by the ectopic expression of CHMP4B-EGFP (Supplementary Fig. 5a–d). In particular, in cells depleted of CHMP4 proteins, partially enveloped primary virions were readily detected in membranous structures in the nucleus (Fig. 3d) and 45.8% of virions in the structures were partially enveloped virions. These virions appeared to be caused by viral budding arrested at the lollipop stage in which nearly complete immature virions remained tethered to the INM. These nearly complete virions were morphologically similar to the virions of other viruses arrested during budding in the absence of ESCRT factors^21^. Similar results were obtained with CHMP4B KO alone (Supplementary Fig. 6), but the effects were less than those with CHMP4B KO in combination with CHMP4A and CHMP4C KD. These results suggest a redundant role for CHMP4 proteins in HSV-1 nuclear egress. In addition, depletion of the CHMP4 protein(s) produced a significant accumulation of nucleocapsids in the cytoplasm (Fig. 3f; Supplementary Table 1; Supplementary Fig. 6e), indicating that ESCRT-III promoted the final envelopment of nucleocapsids in the cytoplasm, as reported previously^22–25^.

In particular, structures similar to the punctate structures with UL34 and the membranous structures observed here were reported to be induced by various mutations in HSV-1 and by KDs of cellular regulators of viral de-envelopment, such as HSV-1 Us3 kinase^4,5,26^. However, in contrast to the observations with depletion of CHMP4 protein(s), the punctate structures with lamin A/C were barely detectable and only 4.4% of virions in the membrane structures were partially enveloped virions in cells infected with the Us3 kinase-dead mutant virus (Supplementary Fig. 7).

### ALIX and VPS4 contribute to HSV-1 nuclear egress

**Fig. 4:**
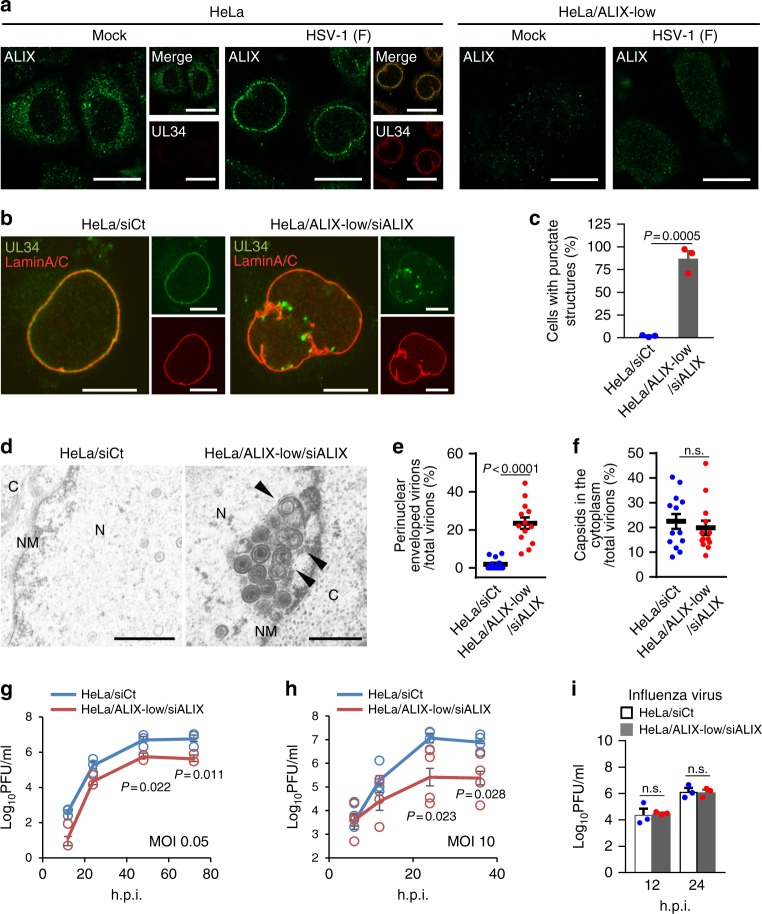
ESCRT-III adaptor protein ALIX contributes to HSV-1 nuclear egress. **a** Confocal microscope images of HeLa or HeLa/ALIX-low cells mock-infected or infected with HSV-1 for 22 h and stained with anti-ALIX and anti-UL34 antibodies. Bars, 20 μm. Images are representative of three independent experiments. **b** Confocal microscope images of HeLa and HeLa/ALIX-low cells treated with control siRNA (siCt) or siRNA to ALIX (siALIX), respectively, for 48 h, and then infected with HSV-1 for 22 h. Bars, 20 μm. Images are representative of three independent experiments. **c** Percent of cells (50–100 cells in each experiment) with aberrant punctate structures along with the nuclear rim in the experiment in (**b**). Data are shown as the mean ± SEM of three independent experiments. **d** Electron microscope images of HeLa and HeLa/ALIX-low cells treated with siCt or siALIX, respectively, for 48 h and infected with HSV-1 for 22 h. C cytoplasm, N nucleus, NM nuclear membrane. Bars, 500 nm. Arrowheads indicate virions defective in the scission steps. Images are representative of three independent experiments. Percent of (**e**) perinuclear enveloped virions and **f** capsids in the cytoplasm of 13 cells in the experiment in (**d**) was determined. Data are shown as the mean ± SEM and are representative of three independent experiments. **g**–**i** HeLa and HeLa/ALIX-low cells treated with siRNA as described in (**b**) were infected with HSV-1 at an MOI of (**g**) 0.05 or (**h**) 10, or with **i** influenza virus at an MOI of 0.01, and progeny virus titers were assayed at the indicated hours post infection (h.p.i.). Data are shown as the mean ± SEM of (**g**, **i**) three or (**h**) four independent experiments. The indicated *P*-values were obtained using the unpaired Student’s *t*-test (**c**, **e**–**i**). n.s., not significant

To investigate the role of an ESCRT-III adaptor protein in HSV-1 nuclear egress, we examined the effect of HSV-1 infection on the cellular localization of ALIX. Although ALIX was predominantly detected in the cytoplasm in mock-infected cells, it was redistributed to the nuclear rim and colocalized with UL34 in HSV-1-infected cells (Fig. 4a). The redistribution of ALIX was dependent on the HSV-1 NEC (Supplementary Fig. 8a–d), similar to that of CHMP4B (Fig. 2).

We then examined the effect of ALIX depletion on HSV-1 nuclear egress. HeLa cells expressing a significantly reduced level of ALIX protein (ALIX-low cells) (Supplementary Fig. 9a, b) were treated with ALIX siRNA to KD ALIX expression further (ALIX-low/KD cells) and were then infected with HSV-1 (Supplementary Fig. 9c, e). In agreement with a previous report^27^, ALIX depletion significantly increased the frequency of multinucleated cells, indicating ALIX depletion reduced the efficiency of cytokinesis (Supplementary Fig. 9d). Depletion of ALIX showed a phenotype similar to that caused by the depletion of CHMP4 protein(s) (Fig. 4b–i), as described above (Fig. 3; Supplementary Fig. 6), i.e., induction of punctate structures at the nuclear rim and membrane invaginations at the INM, accumulation of enveloped virions in the nucleus, and reduced viral replication. Specifically, 18.1% of perinuclear virions were partially enveloped virions in HSV-1-infected ALIX-depleted cells, whereas only 4.4% of virions in the membrane structures were partially enveloped virions in cells infected with the Us3 kinase-dead mutant virus, as described above. However, ALIX depletion had no effect on the accumulation of nucleocapsids in the cytoplasm (Fig. 4f). Similar results were obtained with ALIX-low cells, but the effects were less than those with ALIX-low/KD cells (Supplementary Fig. 10). The effects of ALIX depletion in HSV-1-infected cells were significantly restored by the ectopic expression of ALIX in ALIX-low/KD cells (Supplementary Fig. 11a–d). Notably, it has been reported that the knockdown of ALIX by siRNA had no effect on HSV-1 growth in HeLa cells^24^ and this observation was reproducible in this study (Supplementary Fig. 12). In the current study, the depletion of ALIX in HeLa cells by inactivation of the ALIX gene using the CRISPR/Cas system in combination with siRNA against ALIX or by CRISPR/Cas-mediated ALIX gene inactivation alone significantly reduced HSV-1 replication in the cells. We speculate that this discrepancy between studies might be explained by the different efficiencies of ALIX depletion. These results indicate that ALIX is a regulator of HSV-1 nuclear egress similar to CHMP4 proteins.

**Fig. 5:**
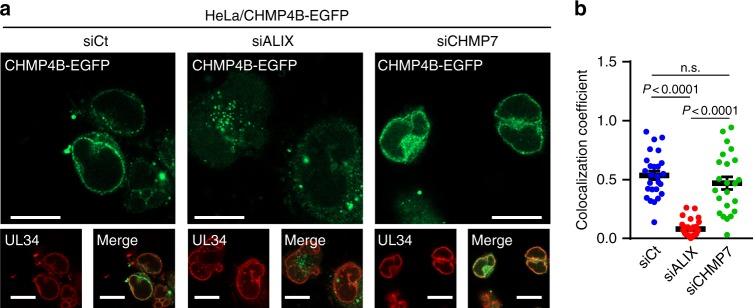
ALIX is required for the recruitment of ESCRT-III to the NM in HSV-1-infected cells. **a** Confocal microscope images of HeLa/CHMP4B-EGFP cells treated with (left) control siRNA (siCt), (middle) siRNA to ALIX (siALIX), or (right) siRNA to CHMP7 (siCHMP7) for 48 h and subsequently infected with HSV-1 for 22 h. Bars, 20 μm. Images are representative of three independent experiments. **b** Colocalization between CHMP4B-EGFP and UL34 in the experiment in (**a**) was quantified using Mander’s colocalization coefficient. Data are shown as the mean ± SEM (*n* = 26 for siCt, 29 for siALIX and 23 for siCHMP7, and are representative of three independent experiments). The indicated *P*-values were obtained using the Tukey’s test. n.s., not significant

**Fig. 6:**
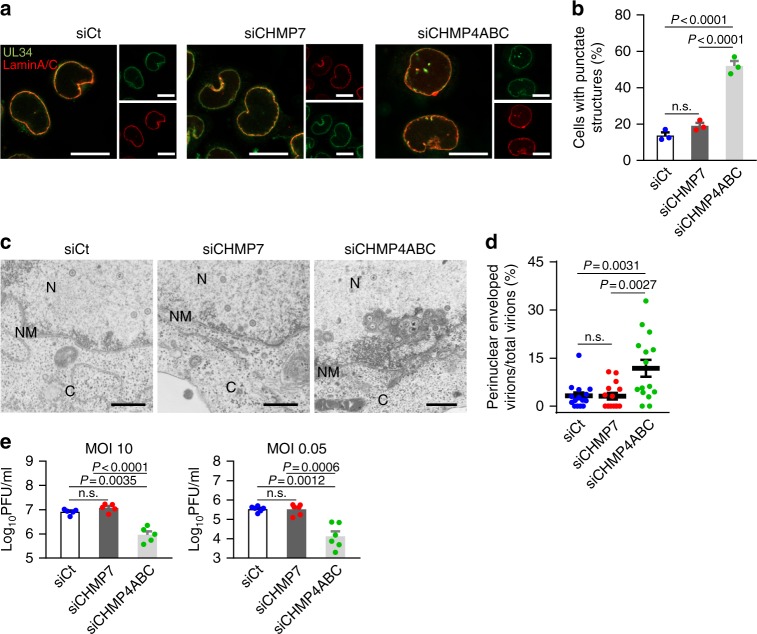
Effects of CHMP7 depletion on HSV-1 replication. **a** Confocal microscope images of HeLa cells treated with control siRNA (siCt), siRNA to CHMP7 (siCHMP7), or siRNA to CHMP4A, B, and C (siCHMP4ABC) for 48 h and infected with HSV-1 for 22 h. Images are representative of three independent experiments. Bars, 20 μm. **b** The percent of cells (100–200 cells in each experiment) with aberrant punctate structures along with the nuclear rim was determined in the experiment in (**a**). Data are shown as the mean ± SEM of three independent experiments. **c** Electron microscope images of HeLa cells treated with siRNA as described in **a** and infected with HSV-1 for 22 h. C cytoplasm, N nucleus, NM nuclear membrane. Bars, 500 nm. Images are representative of three independent experiments. **d** The percent of perinuclear enveloped virions in 15 cells in the experiment in (**c**) was determined. Data are shown as the mean ± SEM and are representative of three independent experiments. **e** HeLa cells treated with siRNA as described in (**a**) were infected with HSV-1 at an MOI of 10 or 0.05 and viral titers were assayed at the indicated times post infection. Data are shown as the mean ± SEM of five (MOI 10) or six independent experiments (MOI 0.05). The indicated *P*-values were obtained using the Tukey’s test (**b**, **d**, **e**). n.s., not significant

To further investigate whether ALIX functions as an adaptor protein(s) for ESCRT-III recruitment during HSV-1 nuclear egress, we examined the effect of ALIX depletion (Supplementary Fig. 13a, b; Supplementary Fig. 14a, c) on the recruitment of CHMP4B to the NM in HSV-1-infected cells. Depletion of ALIX significantly impaired the recruitment of CHMP4B-EGFP and endogenous CHMP4B to the nuclear rim without affecting the accumulation of CHMP4B-EGFP or CHMP4B (Fig. 5a, b; Supplementary Fig. 14d, e). The impairment of CHMP4B-EGFP recruitment by ALIX depletion was restored by the ectopic expression of ALIX (Supplementary Fig. 15). We also examined the effect of the ectopic expression of an ALIX mutant carrying an aspartic acid substitution for isoleucine at ALIX 212 (I212D), which precludes interactions with CHMP4^28^, in ALIX-depleted cells to investigate whether the interaction between CHMP4B and ALIX is required for the recruitment of CHMP4B to the NM. As shown in Supplementary Fig. 15, the ectopic expression of the ALIX mutant did not restore the impairment of CHMP4B-EGFP recruitment in ALIX-depleted cells. Furthermore, because the ESCRT-II/ESCRT-III hybrid protein CHMP7 was proposed to recruit ESCRT-III proteins to the NM during NM reformation^9,14^ and NPC quality control^11^ as described above, we also investigated the effect of CHMP7 depletion on HSV-1 nuclear egress and on the recruitment of CHMP4B to the NM in infected cells. Depletion of CHMP7 (Supplementary Fig. 13; Supplementary Fig. 14a–c) had no obvious effect on the recruitment of CHMP4B-EGFP (Fig. 5a, b) and endogenous CHMP4B (Supplementary Fig. 14d, e), on INM integrity in HSV-1-infected cells and on HSV-1 nuclear egress (Fig. 6a–d) and replication (Fig. 6e), unlike that of CHMP4 and ALIX protein(s).

We also tested whether ALIX interacted with the HSV-1 NEC that is responsible for the recruitment of ESCRT-III to the INM as shown in Fig. 2. ALIX specifically co-precipitated with UL34 tagged with Strep-tag (SE-UL34) from lysates of HeLa cells infected with HSV-1 expressing SE-UL34, whereas CHMP4B did not co-precipitate with SE-UL34 (Supplementary Fig. 16a). In addition, both the purified Bro and V domains of ALIX fused to glutathione *S*-transferase (GST) pulled down UL34 from lysates of HSV-1-infected cells, although GST alone did not (Supplementary Fig. 16b). Collectively, these results indicate that ALIX interacts with the HSV-1 NEC and promotes the recruitment of ESCRT-III to the NM during HSV-1 nuclear egress. Thus, ALIX, but not CHMP7, appears to function as an adaptor in the nucleus for ESCRT-III during HSV-1 nuclear egress, in contrast to recent reports that ESCRT-III-mediated nuclear events were dependent on CHMP7, but not ALIX^9^. Although ALIX functions as an adaptor for various ESCRT-III-mediated cytoplasmic events^7^, HSV-1 appears to utilize other ESCRT-III adaptor(s) for final envelopment in the cytoplasm.

**Fig. 7:**
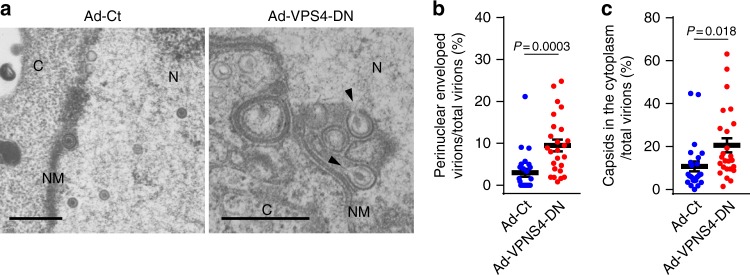
Ectopic expression of the dominant-negative mutant of VPS4 inhibits HSV-1 nuclear egress. **a** Electron microscope images of HeLa cells co-infected with HSV-1 and either control recombinant adenovirus (Ad-Ct) or recombinant adenovirus expressing Flag-VPS4-DN (Ad-VPS4-DN). Cells were infected with Ad-Ct or Ad-VPS4-DN for 4 h and then co-infected with HSV-1 for 22 h. Arrowheads indicate virions defective in scission steps in the aberrant invagination structures derived from the INM. C cytoplasm, N nucleus, NM nuclear membrane. Bars, 500 nm. Images are representative of three independent experiments. The percent of (**b**) perinuclear enveloped virions and **c** capsids in the cytoplasm of the cells in the experiment in (**a**) were determined. Data are shown as the mean ± SEM for 25 cells and are representative of three independent experiments. The indicated *P*-values were obtained using the Tukey’s test (**b**, **c**)

To investigate the role of VPS4 AAA-ATPase in HSV-1 nuclear egress, we examined the effects of a VPS4 dominant-negative mutant on HSV-1 nuclear egress. HeLa cells were co-infected with HSV-1 and a recombinant adenovirus expressing a dominant-negative VPS4 mutant (VPS4-DN) fused to a Flag-tag. In agreement with results for the depletion of CHMP4 protein(s), the ectopic expression of VPS4-DN produced a significant accumulation of enveloped virions in the nucleus and cytoplasm (Fig. 7a–c), and induced membranous invagination structures containing virions that were partially enveloped and tethered to the INM adjacent to the nuclear rim and in the nucleus (Fig. 7a). These results confirmed the significance of the ESCRT-III machinery in HSV-1 nuclear egress.

### ESCRT-III regulates nuclear egress of *Drosophila* RNPs

**Fig. 8:**
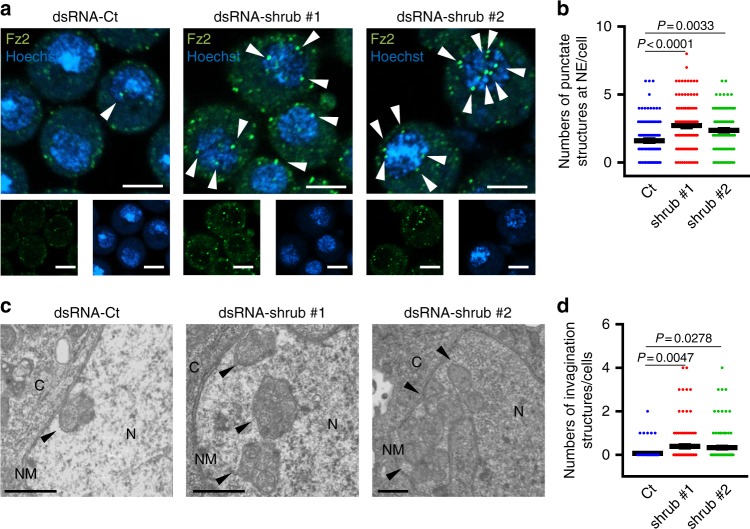
ESCRT-III is required for efficient vesicle-mediated nucleocytoplasmic transport in *Drosophila* S2 cells. **a** Confocal microscope images of S2 cells treated with control dsRNA (dsRNA-Ct) or dsRNA to shrub (dsRNA-shrub), the *Drosophila* ortholog of mammalian CHMP4. Arrowheads indicate the Fz2 foci associated with the nucleus. Bars, 5 μm. Images are representative of three independent experiments. **b** The Fz2-positive punctate structures in dsRNA-treated S2 cells in the experiment in (**a**) were quantified. Data are shown as the mean ± SEM for 100 cells and are representative of three independent experiments. **c** Electron microscope images of S2 cells treated with dsRNA-Ct or dsRNA-shrub. Arrowheads indicate the INM invagination structures with large electron-dense granules. C cytoplasm, N nucleus, NM nuclear membrane. Bars, 500 nm. Images are representative of three independent experiments. **d** The invagination structures with electron-dense granules in dsRNA-treated S2 cells in the experiment in (**c**) were quantified. Data are shown as the mean ± SEM for 101 cells and are representative of 3 independent experiments. The indicated *P*-values were obtained using the Tukey’s test (**b**, **d**)

As described above, the only example of vesicle-mediated nucleocytoplasmic transport in normal (uninfected) cells reported thus far is the nuclear export of RNPs in *Drosophila* cells. In these cells, RNPs are incorporated into large nuclear granules together with *Drosophila* Frizzled 2 (DFz2), which can be detected as DFz2-positive foci by confocal microscopy and as INM invaginations with large electron-dense granules by EM^3,29^. To further confirm the role of ESCRT-III in vesicle-mediated nucleocytoplasmic transport, we examined the effect of the depletion of shrub, a *Drosophila* ortholog of mammalian CHMP4, on the nuclear egress of RNPs in Schneider-2 (S2) cells. The KD of shrub (Supplementary Fig. 17) significantly increased the frequency of DFz2-positive foci associated with the nucleus and the induction of INM invaginations with large electron-dense granules in S2 cells (Fig. 8). These results were in agreement with our conclusion that ESCRT-III regulates the nuclear egress of macromolecular complexes.

### ESCRT-III regulates the integrity of the INM in human cells

**Fig. 9:**
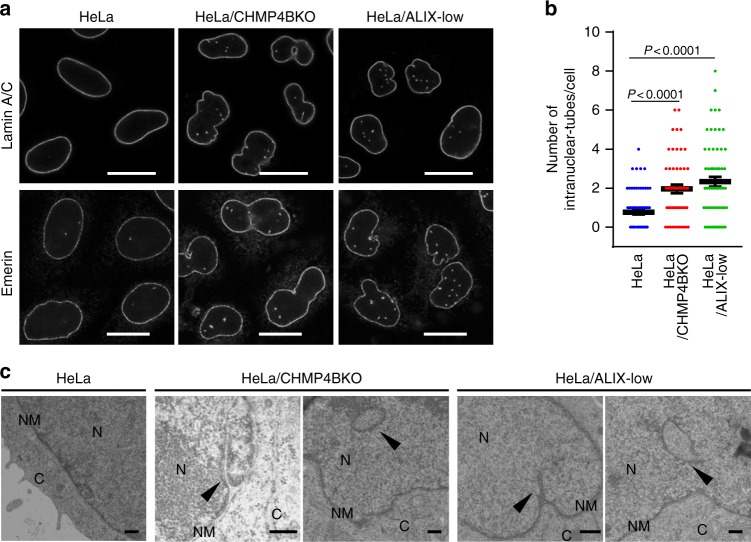
ESCRT-III contributes to maintenance of the integrity of the inner nuclear membrane in normal HeLa cells. **a** Confocal microscope images of HeLa, HeLa/CHMP4B KO, and HeLa/ALIX-low cells stained with Lamin A/C (top row) or Emerin (bottom row). Bars, 20 μm. Images are representative of 3 independent experiments. **b** The number of trans-nuclear tubes in the experiments in (**a**) was measured (*n* = 74 for HeLa, *n* = 55 or HeLa/CHMP4B KO, *n* = 63 for HeLa/ALIX-low). Data are shown as the mean ± SEM and are representative of three independent experiments. The indicated *P*-values were obtained using the Tukey’s test. **c** Electron microscope images of the cells in (**a**). Arrowheads indicate trans-nuclear tubes derived from the INM. C cytoplasm, N nucleus, NM nuclear membrane. Bars, 500 nm. Images are representative of three independent experiments

Finally, we investigated the effect of the ESCRT-III machinery on the INM in uninfected human cells. As shown in Fig. 9, the depletion of CHMP4B or ALIX in HeLa cells significantly induced tube-like structures of lamin A/C and INM proliferation. The induction of lamin A/C-associated tube-like structures observed by the depletion of CHMP4B and ALIX was significantly restored by the ectopic expression of CHMP4B-EGFP and ALIX, respectively (Supplementary Fig. 18). Notably, the depletion of CHMP7 also induced lamin A/C-associated tube-like structures at the nuclear rim (Supplementary Fig. 19a, b). Treatment with Ro3306, an inhibitor of cyclin-dependent kinase 1 (CDK1) that causes cell cycle arrest at the G2/M boundary^30^, significantly inhibited the induction of lamin A/C-associated tube-like structures in CHMP7-depleted cells, but had little effect in CHMP4B- or ALIX-depleted cells (Supplementary Fig. 19c). These results indicate that ESCRT-III downregulates the INM proliferation in normal HeLa cells using a mechanism both dependent and independent of the cell cycle. These observations suggest that ESCRT-III controls the INM integrity in uninfected human cells.

## Discussion

Purified herpesvirus NECs have been reported to be necessary and sufficient for both the deformation and scission of synthetic liposomes in vitro^31^. In addition, recent reports based on the analysis of crystal structures and cryo-imaging of NECs indicated that membrane curvature can be caused by the oligomerization of hexagonal blocks of NECs^1,32^. Although NECs have intrinsic capabilities to remodel membranes, it is not known whether NECs themselves sufficiently carry out herpesvirus primary envelopment in herpesvirus-infected cells. In particular, although it was reported that infection with an HSV-1 UL31-null mutant produced a greater than 1000-fold increase in infectious progeny virus titer, it did form plaques in rabbit skin cells^33^. The NM integrity in rabbit skin cells infected by the HSV-1 UL31-null mutant was similar to that in wild-type HSV-1-infected cells^33^, with nucleocapsids detected in the cytoplasm (Supplementary Fig. 20). These observations suggest that the HSV-1 NEC is not absolutely essential for viral nuclear egress and are in agreement with our conclusion that the NEC functions in viral primary envelopment in concert with the ESCRT-III machinery. Similarly, it was reported that purified Ebola virus VP40 alone, similar to the NEC, can carry out membrane budding in an in vitro system^34^, although Ebola virus budding is highly dependent on ESCRT-III machinery^21^.

The current study shows that the HSV-1 NEC is required for the recruitment of CHMP4 proteins and ALIX to the NM in HSV-1-infected cells, and that ALIX interacts with the NEC. We also show that ALIX and its ability to interact with CHMP4 proteins are required for the recruitment of CHMP4B to the NM in HSV-1-infected cells. These results suggest that the HSV-1 NEC interacts with ALIX at the INM site of HSV-1 primary envelopment, and that this interaction mediates the recruitment of CHMP4 proteins to the INM, similar to HIV-1 GAG p6 and CEP55 in HIV-1 budding and cytokinesis, respectively^8^. ALIX is a well-known adaptor for the recruitment of ESCRT-III proteins in various processes in the cytoplasm, including cytokinesis, exosome secretion, plasma membrane repair, and viral budding^8^. Therefore, this is the first report showing that ALIX has functions in ESCRT-III-mediated nuclear events, including the primary envelopment during vesicle-mediated nucleocytoplasmic transport of HSV-1 nucleocapsids, and regulation of INM integrity in uninfected human cells.

As described above, ESCRT-III was reported to regulate the reformation of the NM, including resealing the NM during late anaphase^9,10^ and repairing NM ruptures caused by cell migration through tight interstitial spaces^13,14^. One could argue that the effects of ESCRT-III depletion on the NM in HSV-1-infected and normal cells shown in this study were not the result of defects in HSV-1 nuclear egress and regulation of INM integrity, but rather caused by problems with the reported role of ESCRT-III in post-mitotic resealing of the NM in mammalian cells^9,10^. However, this seems unlikely based on the following observations. (i) Although CHMP7 was reported to be required for recruitment of CHMP4B during the post-mitotic resealing of the NM^9,14^, we showed in this study that CHMP7 was not required for the recruitment of CHMP4B to the INM during HSV-1 nuclear egress. (ii) In contrast, although ALIX was not required for the recruitment of CHMP4B during post-mitotic resealing of the NM^9^, we showed in this study that ALIX was required for the recruitment of CHMP4B to the INM during HSV-1 nuclear egress. (iii) It is well-established that HSV-1 infection efficiently causes cell cycle arrest at the G1/S or G2/M interfaces^35,36^ and, therefore, HSV-1-infected cells cannot enter mitosis. (iv) Unlike HSV-1-infected cells, we showed that CHMP7 in addition to CHMP4B and ALIX were required for the regulation of INM integrity in normal cells. However, we also showed that the effects of CHMP4B and ALIX were independent of mitosis. These observations ruled out the possibility that the effect of ESCRT-III depletion on the NM in these cells was caused by defects in the post-mitotic resealing of the NM. We should note that CHMP7 was recently reported to be required for NM expansion for sealing NPC in yeast^11^. The observations in HSV-1-infected cells described above also eliminated the possibility that the effect of ESCRT-III depletion on the NM was caused by defects in NM expansion for sealing NPC in these cells. In normal cells, however, CHMP7 was required for the regulation of INM. As we showed that the effects of CHMP4B and ALIX were independent of mitosis, and because the involvement of CHMP7 in NM expansion for sealing NPC has not been reported in mammalian cells, these two possibilities may also be less likely to occur in normal cells.

The INM interacts closely with the nuclear lamina that underlies the nucleoplasmic face of the INM. Mutations in genes encoding INM-associated proteins, such as those encoding lamin A/C and Emerin, are associated with various hereditary diseases, including Emery-Dreifuss muscular dystrophy and Hutchinson–Gilford progeria syndrome^37^. At the cell culture level, the typical phenotype of mutations in lamin A/C and Emerin is the accumulation of these proteins, thereby inducing INM proliferation^38,39^. It was reported that the overexpression of various INM-associated proteins, such as lamin A, lamin B2, lamin B receptor, and Emerin, causes aberrant NM proliferation^40–42^. Taken together, these observations suggest that INM integrity, which appears to be regulated by the quantity of INM-associated proteins at the INM, was critical for the maintenance of homeostasis. Therefore, eukaryotic cells must have evolved a mechanism(s) to maintain INM integrity by controlling the proper accumulation of INM-associated proteins. In this study, we present evidence suggesting that the ESCRT-III machinery is one of these mechanisms, and that ESCRT-III downregulated aberrant INM proliferations.

**Fig. 10:**
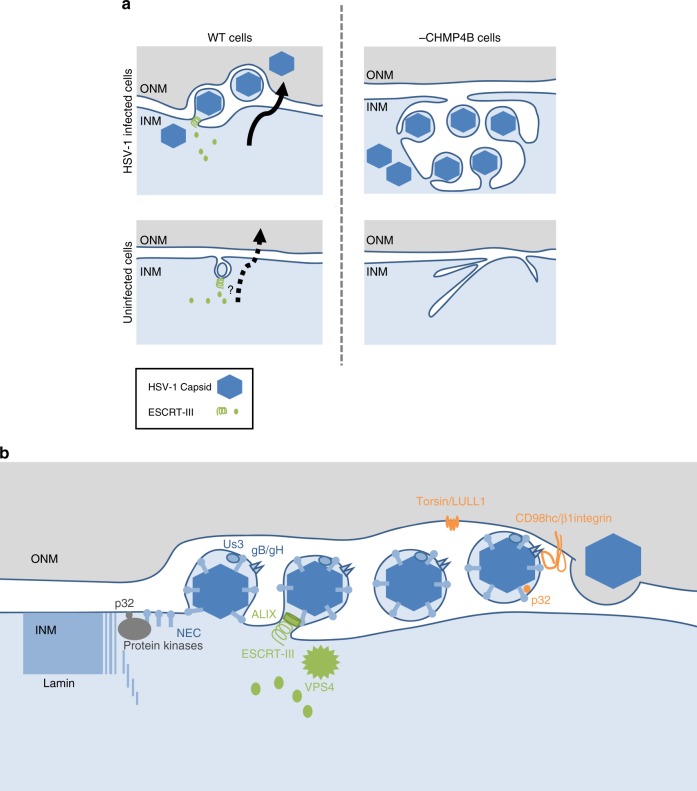
Model for INM scission by ESCRT-III. **a** In HSV-1-infected cells, ESCRT-III is recruited to INM sites, where HSV-1 capsids acquire a primary envelope that functions in INM scission to produce primary enveloped virions in the perinuclear space. Depletion of CHMP4 proteins impairs primary envelopment and produces an accumulation of primary enveloped virions in the invagination structures in the nucleus. Nuclear morphology is maintained by the lamina meshwork but HSV-1 infection dissociates nuclear lamina^6^. Thus, arrested virions might be mainly accumulated in the invagination structures derived from the INM. In normal (uninfected) human cells, ESCRT-III contributes to downregulate excess INM. This process might be similar to the vesicle-mediated nucleocytoplasmic transport of HSV-1 nucleocapsids. CHMP4 KO increases the INM proliferation in a manner independent of cell cycle. **b** Proposed model of vesicle-mediated nucleocytoplasmic transport of HSV-1 nucleocapsids. (i) Protein kinases recruited by the NEC induce local dissolution of the nuclear lamina to allow nucleocapsids access to the INM^6^. Host protein p32 contributes to the recruitment of protein kinase C^65^. (ii) The NEC deforms the INM to wrap around the nucleocapsid. (iii) The NEC recruits ESCRT-III machinery via ALIX and mediates INM scission to complete primary envelopment. (iv) The de-envelopment process is still unclear, but a possible role of viral gB and gH together with host protein CD98hc, β1 integrin, and p32 has been reported^4,5,66^. Phosphorylation of the NEC by the viral Us3 protein kinase promotes de-envelopment^67^. The torsin/LULL1 complex may indirectly contribute to this step^68,69^

We propose a model (Fig. 10) by which ESCRT-III may control INM integrity by regulating the NE budding of excess INM-associated proteins in normal cells. In this model, herpesviruses activate and take over the ESCRT-III-mediated NE budding pathway followed by the de-envelopment process, the precise mechanism of which is not clear, to enable the efficient transmission of viral nucleocapsids from the nucleus to the cytoplasm. These are essential steps in the viral life cycle, because herpesviruses acquire their final envelopes in cytoplasmic vesicles^2^. In support of this model, polymorphisms in the ESCRT-III proteins CHMP2B and CHMP4B are associated with dementia and cataracts, respectively, both of which are associated with mutations in lamin A/C^37,43-45^. It was reported that infection of cells with Epstein–Barr virus (EBV), another herpesvirus, recruited CHMP4B to the NM, as observed in HSV-1-infected cells, and induced vacuole-like structures containing INM-associated proteins in the cytoplasm, unlike HSV-1 infection, and that ESCRT-III was required for the formation of these cytoplasmic structures^46,47^. Although that study did not directly address whether ESCRT-III mediates the nuclear egress of EBV nucleocapsids^46,47^, it supports our conclusion that herpesvirus infection recruits the ESCRT-III machinery to the NM and activates the ability of ESCRT-III to modulate INM integrity.

As described above, ESCRT-III was also reported to be involved in resealing the NM during late anaphase^9,10^ in repairing NM ruptures caused by cell migration through tight interstitial spaces^13,14^ and in sealing the NM for NPC quality control^11,12^. Thus, ESCRT-III appears to regulate various dynamic events in the nucleus by remodeling the NM using different mechanisms, so that cells can: (i) maintain proper integrity of the NM, (ii) establish a physical barrier between the nuclear interior and the cytoplasm after mitosis, (iii) limit DNA damage and cell death caused by NM ruptures, and (iv) maintain NPC through NM sealing.

## Methods

### Cells

HeLa (provided by Dr Shinobu Kitazume)^48^, Rabbit Skin (provided by Dr Bernard Roizman)^49^, MDCK (provided by Dr Hiroomi Akashi)^50^, HEK293 (ATCC, CRL1573)^49^, and Plat-GP (provided by Dr Toshio Kitamura)^51^ cells were maintained in Dulbecco’s modified Eagle’s medium (DMEM) containing 10% fetal calf serum (FCS). Vero cells^49^ were provided by Dr Bernard Roizman and maintained in DMEM medium containing 5% calf serum. UL31-CV1 cells (provided by Dr Joel Baines)^33^ or UL34-Vero cells (generated in our laboratory)^52^ were maintained in DMEM medium containing 10% FCS in combination with 200 μg/ml hygromycin B or 5 μg**/**ml puromycin, respectively. *Drosophila* S2 cells^53^ were provided by Dr Masayuki Miura and cultured in Schneider’s medium containing 10% FCS.

### Viruses

Wild-type HSV-1 (strain F: HSV-1(F)), influenza virus (A/WSN/33; H1N1), recombinant virus HSV-1 Us3K220M encoding an enzymatically inactive Us3 mutant in which lysine at Us3 residue 220 was replaced with methionine, recombinant HSV-1 MEF-UL34 expressing HSV-1 UL34 fused to an MEF tag (Myc epitope-tobacco etch virus [TEV] protease cleavage site-Flag-epitope) with Myc and Flag epitopes and a TEV protease cleavage site (MEF-UL34), recombinant HSV-1 UL31-deletion virus (HSV-1 ΔUL31), recombinant HSV-1 UL34-deletion virus (HSV-1 ΔUL34), and recombinant HSV-1 expressing mRFP (HSV-1-mRFP) were reported previously^50,52,54,55^. HSV-1 ΔUL31 and HSV-1 ΔUL34 were propagated in a UL31-expressing cell line or UL34-expressing cell line, respectively^52,54^. To measure the viral growth of HSV-1(F) and its derivatives, HeLa cells were infected at a multiplicity of infection (MOI) of 10 or 0.05. At the indicated times post infection, total virus from cell culture supernatants and infected cells was collected and assayed on Vero cells. Similarly, cells were infected with wild-type influenza virus at an MOI of 0.01, colected at the indicated times post infection, and assayed on MDCK cells.

### Plasmids

Plasmids pcDNA3.1-UL34 and pcDNA3.1-UL31 used for the expression of HSV-1 UL34 and UL31, respectively, were reported previously^52^. pCHMP4B-EGFP encoding a fusion protein of CHMP4B and EGFP (CHMP4B-EGFP) were constructed by cloning CHMP4B cDNA, amplified by PCR from pCMV(Δ5)-CHMP4B^56,57^ (kindly provided by W. I. Sundquist), into pEGFP-N1 (Clontech, Mountain View, CA) in frame with EGFP. DNA fragments of pCHMP4B-EGFP encoding CHMP4B-EGFP were cloned into pMxs-puro to construct pMxs-CHMP4B-EGFP-puro. pEGFP-CHMP7 was constructed by cloning CHMP7 cDNA into pEGFP-C2 (Clontech) in frame with EGFP. pMxs-ALIX-puro to generate HeLa/ALIX-low/rescue cells was constructed by cloning the PCR-amplified ALIX ORF from pCI-FLAG-ALIX^58^ (kindly provided by W. I. Sundquist) into pMxs-puro. ALIX cDNA was cloned into pcDNA3.1( + ) (Invitrogen, Carlsbad, CA) in frame with a Flag-epitope to generate pcDNA3.1-Flag-ALIX. pcDNA3.1-Flag-ALIX-I212D was constructed by replacing ALIX codons I212 in pcDNA3.1-Flag-ALIX with aspartic acid codons as reported previously^59^. To generate fusion proteins of GST with the ALIX Bro domain (amino acids 1–358) and the ALIX V domain (amino acids 362–702) (GST-ALIX-Bro and GST-ALIX-V, respectively), pGEX-ALIX-Bro and pGEX-ALIX-V were constructed by cloning the appropriate ALIX domain, amplified by PCR from pCI-FLAG-ALIX, and cloned into pGEX-4T-1 (GE Healthcare Bio-Sciences, Piscataway, NJ).

Sense and antisense oligonucleotides were designed for insertion into the *Bbs*I site in the pX330 bicistronic expression vector expressing Cas9 and synthetic single-guide RNA^60^ (Addgene, Cambridge, MA) as follows: 5′-CACCGGGCGGCCCGACCCCCCAGG-3′ and 5′-AAACCCTGGGGGGTCGGGCCGCCC-3′ for CHMP4B, and 5′-CACCGCAGGCCCAGTACTGCCGCG-3′ and 5′-AAACCGCGGCAGTACTGGGCCTGC-3′ for ALIX to produce pX330-CHMP4B and pX330-ALIX, respectively. The DNA oligonucleotides were annealed and incorporated into a pX330 vector linearized with the *Bbs*I restriction enzyme.

Complete list of primers used in this study is available in the Supplementary Table 2.

### Antibodies

Antibodies were purchased and used as follows: commercial mouse monoclonal antibodies against ALIX (sc-53540; Santa Cruz Biotechnology, Dallas, TX; 1:500), Flag (M2, F3165; Sigma, St. Louis, MO; 1:1000), α-tubulin (DM1A, T9026; Sigma; 1:1000), lamin A/C (sc-7292; Santa Cruz Biotechnology; 1:2000), Emerin (4G5, MS-1751-S; Lab Vision Corporation, Fremont, CA; 1:1000), gB (P1105; Virusys Corporation, Taneytown, MD; 1:2000), Strep-tag (4F1, M211-3; MBL, Nagoya, Japan; 1:1000), and DFz2 (12A7; DSHB, Iowa City, IA; 1:6); commercial rabbit polyclonal antibody against GFP (598; MBL; 1:1000), CHMP4B (ab105767; Abcam, Cambridge, UK; 1:1000), CHMP4C (ab155668; Abcam; 1:500), lamin B1 (ab16048-100; Abcam; 1:1000) and calnexin (c4731; Sigma; 1:2000); and commercial mouse polyclonal antibodies against CHMP4A (ab67058; Abcam; 1:500). Mouse polyclonal antibodies against UL31(1:500) and rabbit polyclonal antibodies against UL34 (1:2000) were reported previously^61^.

### Establishment of HeLa cells stably expressing CHMP4B-EGFP

Plat-GP cells, a 293T-derived murine leukemia virus-based packaging cell line, were co-transfected with pMxs-CHMP4B-EGFP-puro and pMDG encoding vesicular stomatitis virus envelope protein G using Lipofectamine 2000 (Invitrogen) according to the manufacturer’s instructions. Supernatants were harvested at 48 h post transfection. HeLa cells then were transduced with the retrovirus-containing supernatants of the transfected Plat-GP cells and selected with 1 μg puromycin**/**ml. Resistant cells transduced by recombinant retrovirus derived from pMxs vectors were cloned from single colonies and designated HeLa/CHMP4B-EGFP.

### Establishment of CHMP4B and ALIX KO HeLa cells

HeLa cells were transfected with pX330-CHMP4B or pX330-ALIX using Lipofectamine 2000 (Invitrogen). At 48 h post transfection, the cells were transferred to a 96-well plate at a density of two cells/well. Further growth of the transfected cells was observed in several wells wherein single colonies were grown. Then, the grown cells were re-seeded in 100-mm diameter dishes and the single colonies were individually picked up for further analysis to identify HeLa/CHMP4B KO and HeLa/ALIX-low cells. To determine the genotypes of each allele from CHMP4B KO and ALIX-low cells, genomic DNA from these cells were amplified by PCR and sequenced directly. In the case of HeLa/CHMP4B KO cells, the sequencing of PCR products showed a single pattern, indicating all alleles had the same mutation (Supplementary Fig. 1h). In the case of HeLa/ALIX-low cells, the sequencing of PCR products showed mixed patterns of sequences, indicating that the sequences of ALIX were variable among the three alleles. Therefore, PCR products were cloned into plasmids and their sequences were determined. As a result, we obtained three patterns of sequences, which represented ALIX sequences of the three alleles of HeLa/ALIX-low cells (Supplementary Fig. 9b).

Plat-GP cells were co-transfected with pMxs-puro, pMxs-CHMP4B-EGFP-puro or pMxs-ALIX-puro, and pMDG. The supernatants were harvested, and HeLa/CHMP4B KO or HeLa/ALIX-low cells were transduced with supernatants from pMxs-CHMP4B-EGFP-puro or pMxs-ALIX-puro, respectively, and selected with 1 μg puromycin**/**ml as described above. Resistant cells were designated HeLa/CHMP4B KO/CHMP4B-EGFP and HeLa/ALIX-low/rescue, respectively. Similarly, HeLa, HeLa/CHMP4B KO, and HeLa/ALIX-low cells were each transduced with supernatants from pMxs-puro, selected with 1 μg puromycin**/**ml and designated HeLa/puro, HeLa/CHMP4B KO/puro and HeLa/ALIX-low/puro cells, respectively.

### KD experiments

Small interfering RNAs (siRNAs) with target sequences to CHMP4A (5′-AAGUAUGGGACCAAGAAUA-3′), CHMP4B (5′-CGAUAAAGUUGAUGAGUUA-3′), CHMP4C (5′-UGGCAGAACUUGAAGAAUU-3′), ALIX (5′-GAACAAAUGCAGUGAUAUA-3′), CHMP7 (5′-AGGUCUCUCCAGUCAAUGA-3′ and (5′-GCAAUAGGCAUUUUACCAA-3′) and a control sequence were purchased from Dharmacon (Lafayette, CO). HeLa cells and their derivatives were treated with 1 nM siRNA for 48 h and then infected with HSV-1(F) for further analysis. For rescue experiments, HeLa/CHMP4B-EGFP cells were treated with 1 nM siRNA to ALIX for 24 h, transfected with ALIX expression vectors or control vectors for a further 6 h and infected with HSV-1(F) for 22 h.

### Characterization of KO and KD cells

For the Multinucleation assay, cells were fixed and stained with anti-α-tubulin antibodies and Hoechst 33342 (Invitrogen). Then, cells were analyzed for the presence of more than one nucleus per cell. Cells connected by midbodies were considered multinucleated. The viability of cells was assayed using a cell counting kit-8 (Dojindo, Tokyo, Japan) according to the manufacturer’s instructions.

### Generation of recombinant HSV-1

Recombinant virus YK540 (SE-UL34) expressing UL34 fused to a Strep-tag and a TEV protease cleavage site was constructed by a two-step Red-mediated mutagenesis procedure^55^ using *Escherichia coli* GS1783 carrying pYEbac102, with the following primers 5′-GAACCCTTTGGTGGGTTTACGCGGGCACGCACGCTCCCATCGCGGGCGCCATGGCTAGCTGGAGCCACCC-3′, 5′-TCGAAGGCGTCACCTGGGTGGCCGGTGTAGGGCTTGCCCAGTCCCGCCATACCCTGAAAATACAAATTCT-3′ and pEP-KanS-SEM as a template.

### Inhibition of the ESCRT-III pathway by a VPS4-DN

Plasmid pcDNA-EF-1αp was constructed by cloning a DNA fragment containing the elongation factor 1α (EF-1α) promoter region, amplified by PCR from pEF-BOS (kindly provided by K. Miyake), into the pcDNA3.1 *Nru*I and *Hin*dIII sites (Invitrogen), resulting in substitution of the human cytomegalovirus promoter region in pCDNA3.1 with the EF-1α promoter. Plasmid pENTR11-EF-1αp was constructed by cloning a DNA fragment containing the EF-1 promoter, multi-cloning sites and the bGH (bovine growth hormone) polyadenylation signal, amplified by PCR from pcDNA-EF-1α, into the pENTR11 *Nco*I and *Eco*RV sites (Invitrogen). A DNA fragment encoding Flag-tagged VPS4-DN, amplified by PCR from pEGFP-VPS4A-EQ (kindly provided by W. I. Sundquist)^62^, encoding VPS4-DN in which glutamate in the VPS4 ATPase active site was replaced with glutamine, was cloned into pENTR11-EF-1αp to yield pENTR11-EF-1αp-VPS4-DN. To generate pAd/PL-DEST-EF-1αp and pAd/PL-DEST-EF-1αp-VPS4-DN, LR reactions with pAd/PL-DEST in combination with pENTR11-EF-1αp or pENTR11-EF-1αp-VPS4-DN, respectively, were performed using the LR Clonase Enzyme Mix (Invitrogen). HEK293 cells were transfected with pAd/PL-DEST-EF-1αp or pAd/PL-DEST-EF-1αp-VPS4-DN using Lipofectamine 2000, and progeny recombinant adenoviruses were collected and designated Ad-Ct and Ad-Flag-VPS4-DN, respectively. Cells were infected with Ad-Ct or Ad-VPS4-DN at an MOI of 10 for 4 h and then co-infected with HSV-1 for further analysis.

### Affinity precipitation

HeLa cells were incubated with wild-type HSV-1(F) or YK540 expressing SE-UL34 at an MOI of 0.1 for 48 h, collected, and lysed with 0.5% NP-40 buffer (50 mM Tris-HCl [pH 8.0], 150 mM NaCl, 0.5% NP-40) containing a protease inhibitor cocktail (Nacalai Tesque, Kyoto, Japan). After centrifugation, the supernatants were reacted with StrepTactin Sepharose beads (IBA, Goettingen, Germany) with rotation for 2 h at 4 °C. The precipitates were collected by brief centrifugation, washed extensively with 0.5% NP-40 buffer, and analyzed by immunoblotting.

### GST-pull down

GST, GST-ALIX-Bro, and GST-ALIX-V were each expressed in *E. coli* and purified on glutathione-sepharose beads. Vero cells infected with MEF-UL34 at an MOI of 0.1 for 48 h were lysed in 0.5% NP-40 buffer containing a protease inhibitor cocktail and incubated with purified GST proteins immobilized on glutathione-sepharose beads (GE Healthcare Bio-Sciences) for 2 h at 4 °C with rotation. Then, the beads were washed extensively with 0.5% NP-40 buffer and analyzed by immunoblotting^50^.

### *Drosophila* S2 cell culture and dsRNA treatment

*Drosophila* S2 cells were treated with dsRNA as follows^53^. Control and shrub dsRNAs were prepared by amplifying pBluescript KS( + ) or S2 cell cDNAs, respectively, by PCR using primers 5′-TAATACGACTCACTATAGGTAAATTGTAAGCGTTAATATTTTG-3′ and 5′-TAATACGACTCACTATAGGAATTCGATATCAAGCTTATCGAT-3′ for control dsRNA, 5′-TAATACGACTCACTATAGGGATGATCCAGACATGAAG-3′ and 5′-TAATACGACTCACTATAGGGTCGATACAAAGCTAAGACTG-3′ for shrub#1 dsRNA, and 5′-TAATACGACTCACTATAGGGGGAAGATGTTCGGCGGCAAG-3′ and 5′-TAATACGACTCACTATAGGGGTTCTCCTGCTCCAGCTCGT-3′ for shrub#2 dsRNA. In vitro transcription was carried out using the T7 RiboMAX Express RNAi System (Promega, Fitchburg, Wisconsin). For dsRNA treatment, 10 μg dsRNA was added to S2 cells cultured in 12-well plates every 2 days for 6 days. At 5 days after the first dsRNA treatment, S2 cells were collected for further analysis. Semi-quantitative RT-PCR analysis was carried out as follows. Total RNA was extracted from dsRNA-treated S2 cells with a High Pure RNA Isolation Kit (Roche, Penzberg, Germany), and cDNA was synthesized from the isolated RNA with a Transcriptor First Strand cDNA Synthesis Kit (Roche) according to the manufacturer’s instructions. Equal amounts of shrub and glyceraldehyde-3-phosphate dehydrogenase (GAPDH) cDNAs were amplified using the following primers: 5′-ATGAGTTTCTTCGGGAAGAT-3′ and 5′-TTAGTTGGACCAGGATAAAA-3′ for shrub and 5′-ACTCGACTCACGGTCGTTTC-3′ and 5′-GCCGAGATGATGACCTTCTT-3′ for GAPDH^29^.

### Immunoblotting and immunofluorescence

For immunoblotting, cell lysates in SDS sample buffer (62.5 mM Tris-HCl [pH 6.8], 20% glycerol, 2% SDS, 5% 2-mercaptoethanol) were subjected to electrophoresis in denaturing gels, transferred to nitrocellulose or polyvinylidene difluoride membranes. The membranes were blocked with 5% skim milk in T-PBS (phosphate-buffered saline (PBS) containing 0.05% Tween 20) for 30 min and reacted with indicated antibodies at least 1 h at room temperature or 4 °C. These membranes were then reacted with secondary antibodies conjugated with peroxidase (GE Healthcare Bio-Sciences) and visualized using ECL (GE Healthcare Bio-Sciences) with ImageQuant LAS 4000 (GE Healthcare Bio-Sciences). For immunofluorescence, either (i) HeLa cells and their derivatives were infected with HSV-1 at an MOI of 10 for 22 h, or (ii) HeLa cells and their derivatives were transfected with the indicated expression vectors for 24 h, or (iii) HeLa cells were transfected with the indicated expression vectors for 6 h and then infected with HSV-1-mRFP at an MOI of 10 for 22 h, or (iv) *Drosophila* S2 cells were treated with dsRNA for 5 days. The cells were fixed with 4% paraformaldehyde for 10 min, permeabilized with 0.1% Triton-X100 for 5 min, and blocked with PBS containing 10% human serum for 30 min. These cells were reacted with indicated antibodies for 1 h at room temperature, followed by reaction with secondary antibodies conjugated to Alexa Fluor (Invitrogen) for 1 h at room temperature. Then, the cells were examined with a Zeiss LSM 5 or LSM800 microscope (Zeiss, Oberkochen, Germany). Hoechst 33342 was used for nuclear staining. For super-resolution imaging, HeLa-CHMP4B-EGFP cells were infected with HSV-1 at an MOI of 10 for 22 h, and then fixed and stained with the indicated antibodies as described above. Image acquisition was performed in the two-dimensional structured illumination microscopy (SIM) mode with a Nikon microscope (N-SIM; Nikon, Tokyo, Japan), and image reconstruction was carried out using NIS-Elements software (Nikon)^63^. To measure colocalization, images acquired by the LSM800 microscope (Zeiss, Oberkochen, Germany) were analyzed with the colocalization function in ZEN2.1 software (Zeiss) as previously reported^52^. Briefly, regions of the image that did not contain a visual signal were selected and used to threshold the image. Mander’s colocalization coefficient (*M*) was calculated using the following equation, as reported previously^52^: *M* = Σ*iXi*.colocalized/Σ*iXi*, where *Xi* is equal to the intensity of marker *X* at a pixel and *Xi*.colocalized is the intensity of the pixels where the intensity of the other marker is greater than the threshold value. An *M*-value of 1.0 indicates 100% colocalization and 0 indicates 0% colocalization.

### Ro3306 treatment

HeLa cells were treated with 1 nM siRNA for 24 h and treated with 10 μM Cdk1 inhibitor (Ro3306) for another 24 h. Then, the cells were fixed, permeabilized and stained with anti-lamin A/C antibody for immunofluorescence analysis.

### Electron microscopy

For these studies, either (i) HeLa cells and their derivatives were infected with wild-type HSV-1 at an MOI of 20 for 22 h, (ii) rabbit skin cells were infected with HSV-1 ΔUL31 at an MOI of 3 for 18 h, or (iii) *Drosophila* S2 cells were treated with dsRNA for 5 days. The cells were fixed with 2% paraformaldehyde and 1% glutaraldehyde overnight at 4 °C, post-fixed with 2% osmium tetroxide on ice for 2 h, washed with distilled water, dehydrated with an ethanol gradient series, incubated in propylene oxide, and embedded in an Epon 812 resin mixture. Then, these samples were sectioned on grids, stained with 2% uranyl acetate and Reynold’s lead citrate and examined by transmission EM^61^. Immunoelectron microscopy was performed as follows^61,64^. HeLa-CHMP4B-EGFP cells infected with HSV-1 at an MOI of 20 for 24 h were fixed with 2% paraformaldehyde and 1% glutaraldehyde on ice for 2 h, post-fixed with 2% osmium tetroxide on ice for 2 h, washed with distilled water, dehydrated with an ethanol gradient series, incubated in propylene oxide, and embedded in an Epon 812 resin mixture. Ultrathin sections were prepared as described above. After a PBS wash, the sections were incubated with 10% human serum and 5% bovine serum albumin in PBS and then with anti-GFP rabbit polyclonal antibody. The sections were then washed with PBS and incubated with goat anti-mouse IgG conjugated to 10 nm gold particles. After immunostaining, the sections were stained as described above and examined by transmission EM. For statistical analyses, the length of NM was calculated by ImageJ software (NIH, Bethesda, MD).

### Statistical analysis

For the comparison of two groups, statistical analysis was performed using the unpaired Student’s *t*-test. Tukey’s test was used for multiple comparisons. A *P*-value > 0.05 was considered not significant (n.s.). All statistical analysis was performed in GraphPad Prism 7 (GraphPad Software, San Diego, CA). No methods were used to determine whether the data meet assumptions of the statistical approach.

## Electronic supplementary material

Supplementary information

## Data Availability

The authors declare that the data supporting the findings of this study are available within the article and its Supplementary Information files, or are available on request.
