# Supplementary material for: ESCRT-III mediates budding across the inner nuclear membrane and regulates its integrity
Source: Nat Commun. 2018 Aug 23;9:3379. doi: 10.1038/s41467-018-05889-9 (PMC6107581; doi:10.1038/s41467-018-05889-9)
Supplement: Supplementary file 1 — Supplementary information [file 41467_2018_5889_MOESM1_ESM.pdf]

Supplementary information

ESCRT-III mediates budding across the inner nuclear membrane and regulates its integrity

Arii et al.

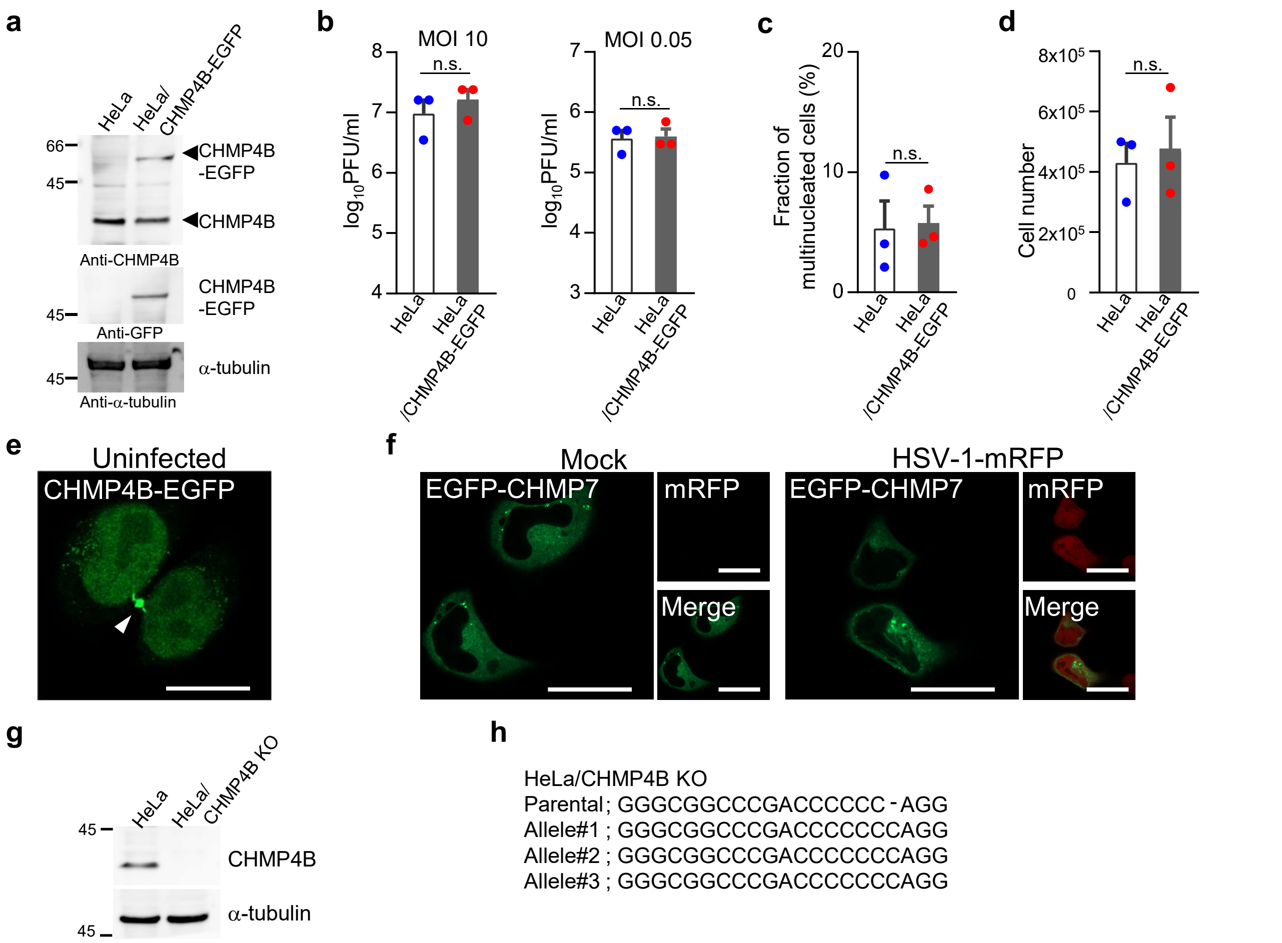

Supplementary Figure 1. Characterization of HeLa/CHMP4B-EGFP and HeLa/CHMP4B KO cells. (**a**) Lysates of HeLa and HeLa/CHMP4B-EGFP cells were analyzed by immunoblotting with anti-CHMP4B, anti-GFP and anti-α-tubulin antibodies. CHMP4B-EGFP was expressed at a level comparable to that of endogenous CHMP4B in HeLa/CHMP4B-EGFP cells. Images are representative of 3 independent experiments. (**b**) HeLa and HeLa/CHMP4B-EGFP cells were infected with HSV-1 at an MOI of 10 or 0.05, harvested at 24 h or 48 h post-infection, respectively, and virus titers were assayed on Vero cells. Data are shown as the mean ± s.e.m. of 3 independent experiments. HSV-1 grew as well in HeLa/CHMP4B-EGFP cells as in HeLa cells. (**c**) Stable expression of CHMP4B-EGFP does not inhibit cytokinesis. HeLa or HeLa/CHMP4B-EGFP cells were fixed, stained with both anti-α-tubulin antibodies and Hoechst 33324, and scored for multinucleated cells. Overall, 100-300 cells in each experiment were analyzed for the presence of more than one nucleus per cell. Data are shown as the mean ± s.e.m of 3 independent experiments. (**d**) Stable expression of CHMP4B-EGFP does not affect cell proliferation. 10^5 of HeLa or HeLa/CHMP4B-EGFP cells were plated into each well of a 24-well plate. At 48 h later, cells were collected and counted. Data are shown as the mean ± s.e.m of 3 independent experiments. (**e**) CHMP4B-EGFP localized in the midbody-like structure during cytokinesis. HeLa/CHMP4-EGFP cells were analyzed by confocal microscopy. Arrowhead indicates midbody-like localization of CHMP4B-EGFP. Bars, 20 μm. Image is representative of 3 independent experiments. (**f**) HeLa cells were transfected with the EGFP-CHMP7 expression vector for 6 h and infected with HSV-1-mRFP for 22 h, and then analyzed by confocal microscopy. Bars, 20μm. Images are representative of 3 independent experiments. (**g**) Lysates of HeLa and HeLa/CHMP4B KO cells were analyzed by immunoblotting with anti-CHMP4B and anti-α-tubulin antibodies. Images are representative of 3 independent experiments. (**h**) The targeted CHMP4B mutation sequences and the parental sequence in HeLa/CHMP4B KO cells are shown. The indicated P values were obtained using the unpaired Student's t-test (**b-d**). n.s., not significant.

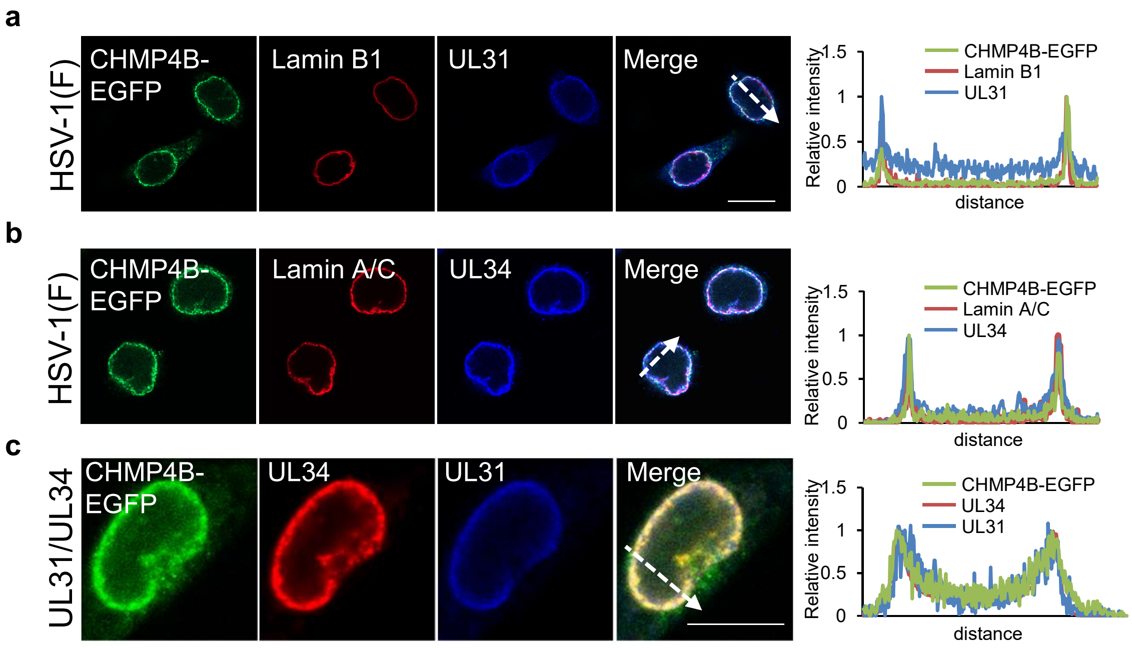

Supplementary Figure 2. HSV-1 NEC proteins co-localize with CHMP4B-EGFP at the nuclear rim. Fluorescence line scans along the dotted lines of confocal microscope images of HeLa/CHMP4B-EGFP cells infected with wild-type HSV-1 (**a, b**) or transfected with the UL31 and UL34 expression vectors (**c**) are shown on the right of each image. The images in a, b and c are identical to those shown in Fig. 2a, Fig. 2c and Fig. 2e, respectively.

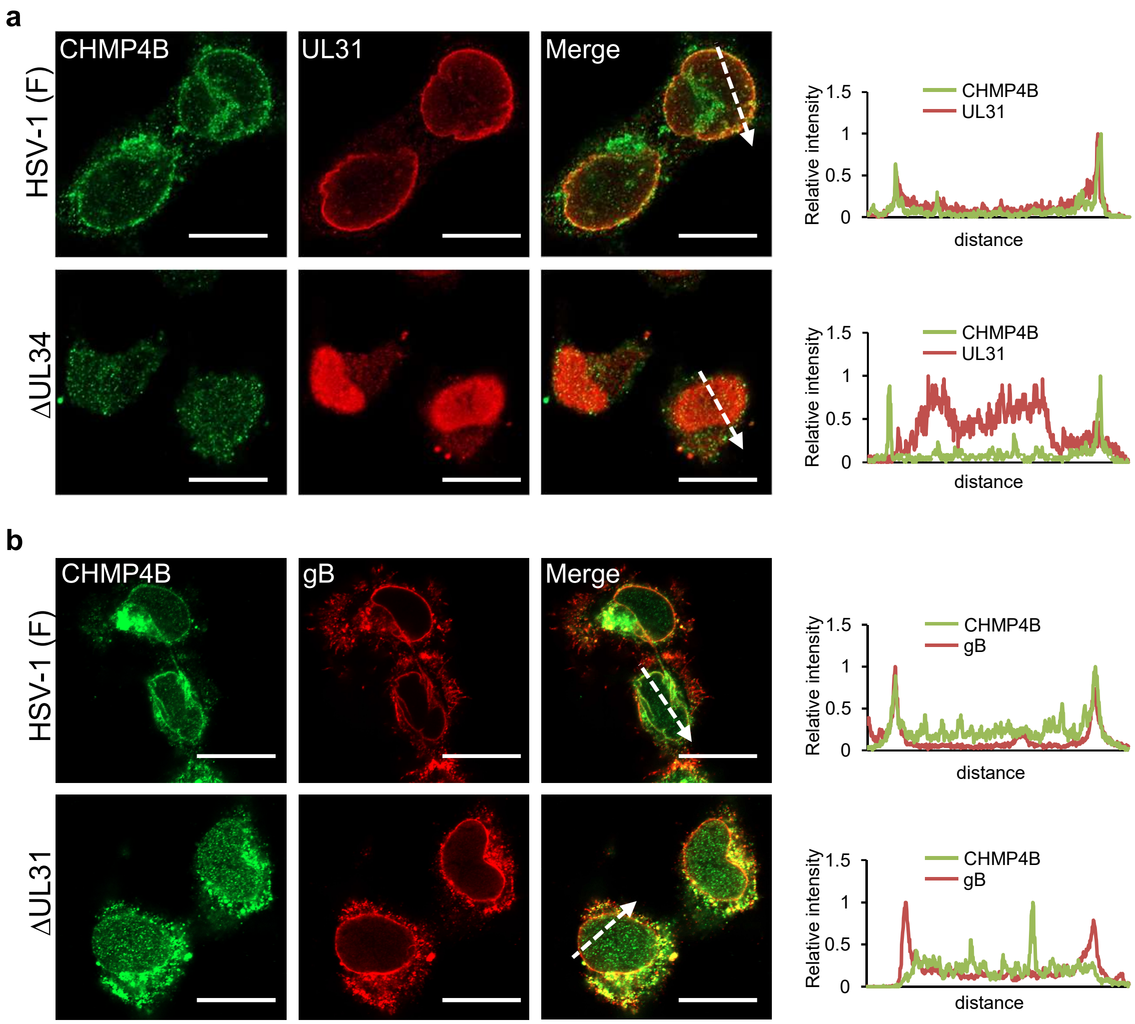

Supplementary Figure 3. HSV-1 NEC regulates the recruitment of endogenous CHMP4B to the nuclear rim in HSV-1-infected cells. (**a**) Confocal microscope images of HeLa cells infected with wild-type HSV-1 or HSV-1 ΔUL34 for 22 h and stained with anti-CHMP4B and anti-UL31 antibodies. Fluorescence line scans along the dotted lines of confocal microscope images are shown on the right of each image. Images are representative of 3 independent experiments. (**b**) Confocal microscope images of HeLa cells infected with wild-type HSV-1 or HSV-1 ΔUL31 for 22 h and stained with anti-CHMP4B and anti-gB antibodies. Bars, 20μm. Fluorescence line scans along the dotted lines of confocal microscope images are shown on the right of each image. Images are representative of 3 independent experiments..

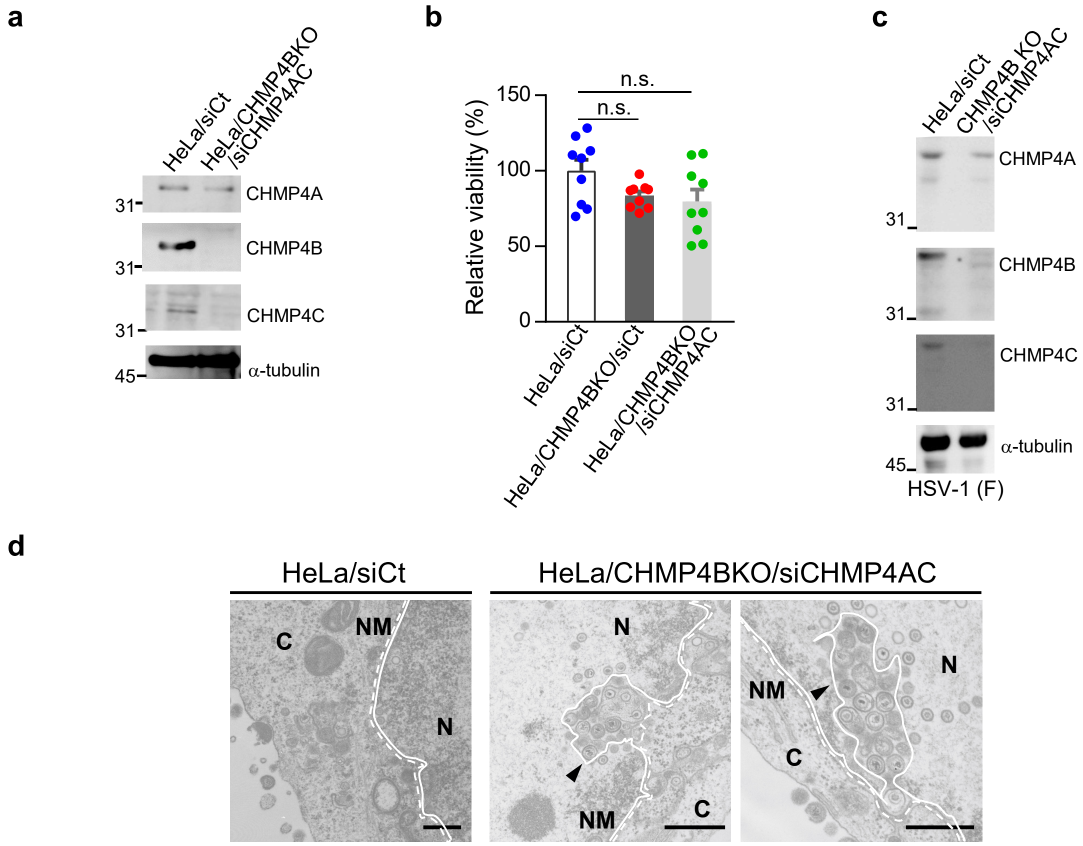

Supplementary Figure 4. Effects of CHMP4 depletion in HSV-1-infected cells. (**a**) Lysates of HeLa and HeLa/CHMP4B KO cells treated with control siRNA (siCt) or siRNAs to CHMP4A and CHMP4C (siCHMP4AC), respectively, for 48 h were analyzed by immunoblotting with anti-CHMP4A, anti-CHMP4B, anti-CHMP4C and anti-α-tubulin antibodies. The expression levels of CHMP4A, CHMP4B and CHMP4C in HeLa/CHMP4B KO cells treated with siCHMP4AC were lower than the levels in HeLa cells treated with siCt. Images are representative of 3 independent experiments. (**b**) The cell viability of HeLa and HeLa/CHMP4B KO cells treated with siCt or siCHMP4AC, respectively, for 48 h. Data are shown as the mean ± s.e.m. of triplicate experiments from 3 independent experiments and are expressed relative to the mean for HeLa cells treated with control siRNA, which was normalized to 100%. The indicated P values were obtained using the Tukey's test (n.s., not significant). (**c**) Lysates of HeLa and HeLa/CHMP4B KO cells treated with siCt or siCHMP4AC, respectively, for 48 h and infected with HSV-1 for 22 h, were analyzed by immunoblotting with the indicated antibodies. Images are representative of 3 independent experiments. (**d**) HeLa and HeLa/CHMP4B KO cells were treated with siCt or siCHMP4AC, respectively, for 48 h, infected with HSV-1 for 22 h and examined by electron microscopy. N, nucleus; C, cytoplasm; NM, nuclear membrane. Straight lines and dotted lines indicate the INM and ONM, respectively. Bars, 500 nm. Arrowheads indicate invagination structures derived from the INM. Images are representative of 3 independent experiments.

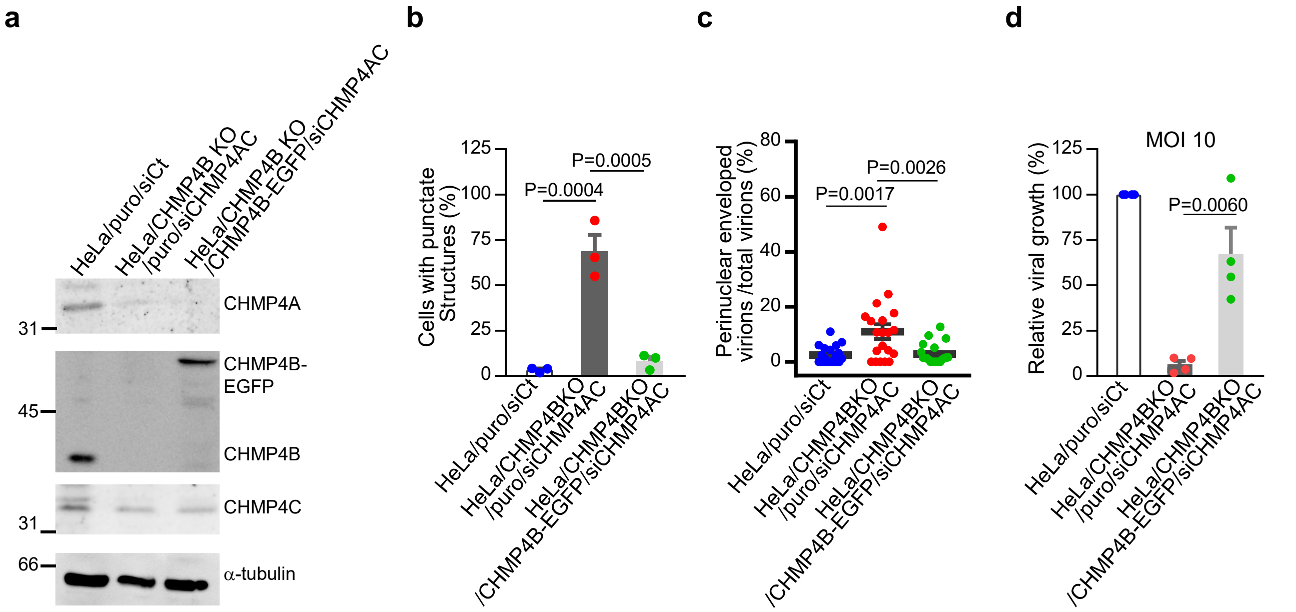

Supplementary Fig. 5. Effect of ectopic expression of CHMP4B-EGFP in CHMP4-depleted and HSV-1-infected cells. (**a**) Lysates of HeLa/puro cells were treated with control siRNA (siCt) for 48 h, and HeLa/CHMP4B KO/puro and HeLa/CHMP4B KO/CHMP4B-EGFP cells were treated with siRNAs to CHMP4A and CHMP4C (siCHMP4AC) for 48 h and analyzed by immunoblotting with anti-CHMP4A, anti-CHMP4B, anti-CHMP4C and anti-α-tubulin antibodies. Images are representative of 3 independent experiments. (**b**) HeLa/puro, HeLa/CHMP4B KO/puro and HeLa/CHMP4B KO/CHMP4B-EGFP cells were treated with siRNAs as described in (**a**) and infected with HSV-1 for 22 h. The percent of cells (100-200 cells in each experiment) with aberrant punctate structures along with the nuclear rim was determined. Data are shown as the mean ± s.e.m. and are representative of 3 independent experiments. (**c**) HeLa/puro, HeLa/CHMP4B KO/puro and HeLa/CHMP4B KO/CHMP4B-EGFP cells were treated with siRNAs as described in (**a**) and infected with HSV-1 for 22 h. The percent of perinuclear enveloped virions in 20 cells observed by EM, as described for Fig. 3c, d, was determined. Data are shown as the mean ± s.e.m. and are representative of 3 independent experiments. (**d**) HeLa/puro, HeLa/CHMP4B KO/puro and HeLa/CHMP4B KO/CHMP4B-EGFP cells were treated with siRNAs as described in (**a**), infected with HSV-1 at an MOI of 10 and progeny virus titers were assayed at 24 h post-infection. Data from 4 independent experiments are shown as the mean ± s.e.m. of the relative titer compared to HeLa/puro cells treated with control siRNA. The indicated P values were obtained using the Tukey's test (**b**, **c**), or the unpaired Student's t-test (**d**).

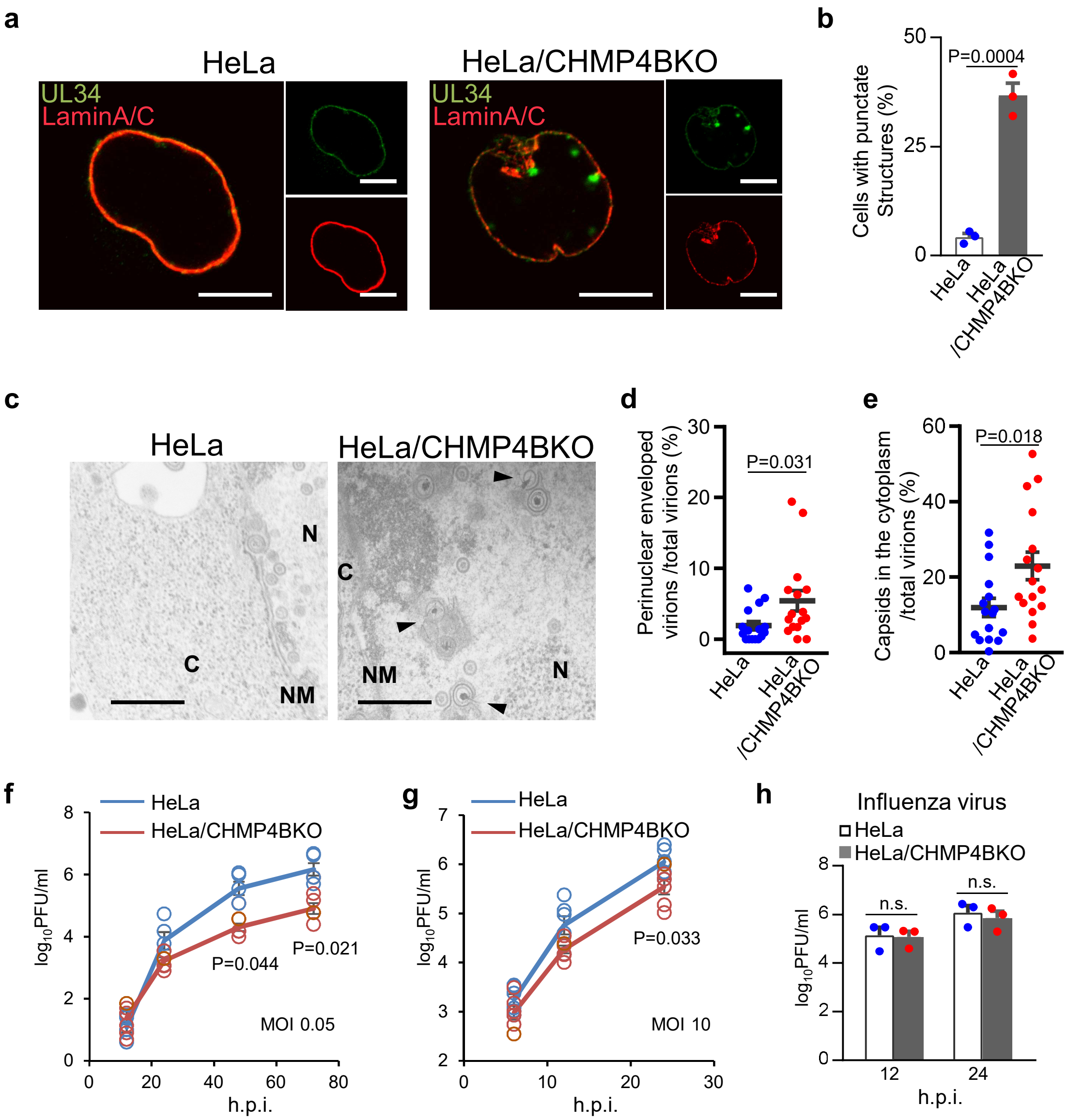

Supplementary Figure 6. Effects of CHMP4B KO in HSV-1-infected cells. (**a**) Confocal microscope images of HeLa cells and HeLa/CHMP4B KO cells, infected with HSV-1 for 22 h and stained with anti-lamin A/C and anti-UL34 antibodies. Images are representative of 3 independent experiments. Bars, 20 μm. (**b**) The percent of cells (50-150 cells in each experiment) with aberrant punctate structures along with the nuclear rim in the experiment described in (**a**) was determined. Data are shown as the mean ± s.e.m. of 3 independent experiments. (**c**) Electron microscope images of HeLa and HeLa/CHMP4B KO cells infected with HSV-1 at an MOI of 20 for 22 h. N, nucleus; C, cytoplasm; NM, nuclear membrane. Bars, 500 nm. Arrowheads show virions defective in the scission steps in the aberrant invagination structures derived from the INM. Images are representative of 3 independent experiments. The percent of (**d**) perinuclear enveloped virions and (**e**) capsids in the cytoplasm in 16 cells was determined. Data are shown as the mean ± s.e.m. and are representative of 3 independent experiments. (**f-h**) HeLa and HeLa/CHMP4B KO cells were infected with HSV-1 at an MOI of (**f**) 0.05 or (**g**) 10, or (**h**) with influenza virus at an MOI of 0.01. At the indicated hours post-infection (h.p.i.), viral growth was assayed. Data are shown as the mean ± s.e.m. of (**f**) 5, (**g**) 6 and (**h**) 3 independent experiments. The indicated P values were obtained using the unpaired Student's t-test (**b, d-h**). n.s., not significant.

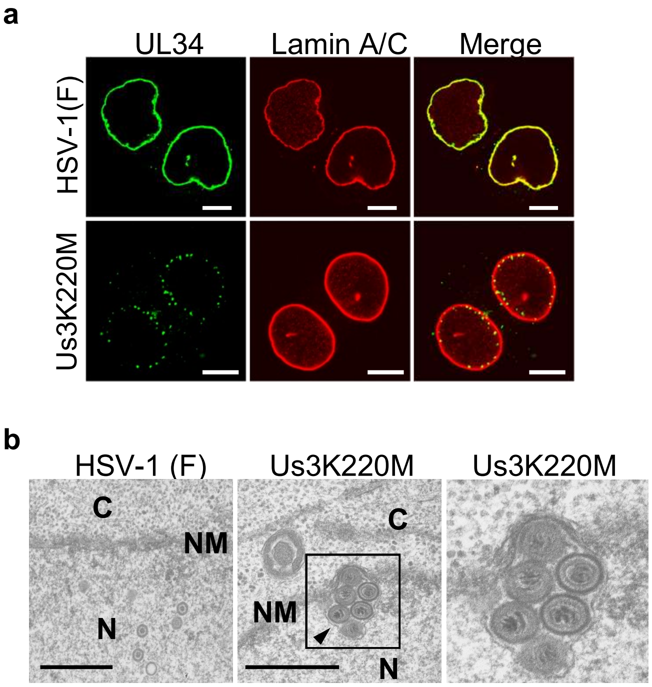

Supplementary Figure 7. Effects of HSV-1 Us3 protein kinase in HSV-1-infected cells. (**a**) Confocal microscope images of HeLa cells infected with wild-type HSV-1 or the Us3 kinase-dead mutant virus (Us3K220M) for 22 h and stained with anti-UL34 and anti-lamin A/C antibodies. Images are representative of 3 independent experiments. (**b**) Electron microscope images of HeLa cells infected with wild-type HSV-1 or the Us3 kinase-dead mutant virus (Us3K220M) for 22 h. The right image is a magnified image of the boxed area in the middle image. Rate of primary enveloped virions arrested at the scission step were rarely detected (4.4 $\pm$ 0.11%) in the invagination structures in cells infected with the Us3K220M mutant virus, in contrast to cells with decreased ESCRT-III. Images are representative of 3 independent experiments. N, nucleus; C, cytoplasm; NM, nuclear membrane. Bars, 500 nm. The arrowhead indicates an invagination structure containing primary enveloped virions.

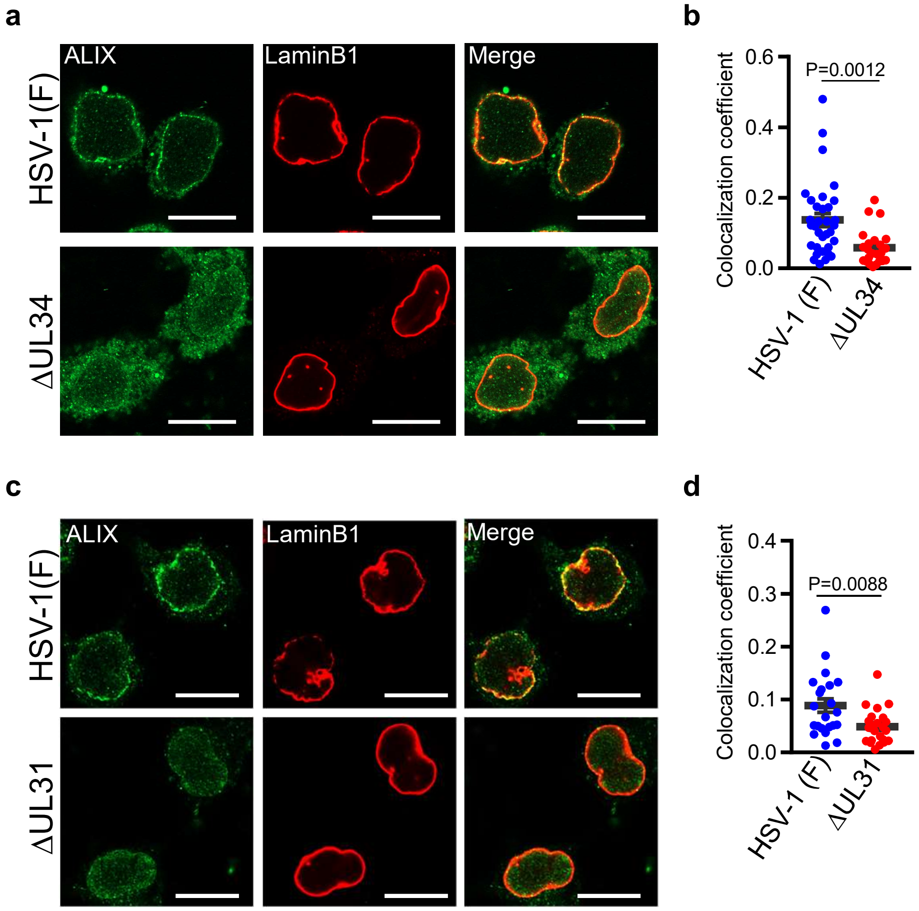

Supplementary Figure 8. HSV-1 NEC is required for the recruitment of ALIX to the NM in HSV-1 infected cells. (**a, c**) Confocal microscope images of HeLa cells infected with wild-type HSV-1 or HSV-1 ΔUL34 (**a**) or HSV-1 ΔUL31 (**c**) for 22 h and stained with anti-ALIX and anti-LaminB1 antibodies. Images are representative of 3 independent experiments. Bars, 20 µm. (**b, d**) Co-localization between ALIX and LaminB1 was quantified using Mander's colocalization coefficient in the experiment in (**a, c**). Data are shown as the mean ± s.e.m. (n=33 for wild-type HSV-1 or 24 for HSV-1 ΔUL34-infected cells in (**c**) and n=22 for wild-type HSV-1 or 23 for HSV-1 ΔUL31-infected cells in (**d**) and are representative of 3 independent experiments. The indicated P values were obtained using the unpaired Student's t-test (**b, d**).

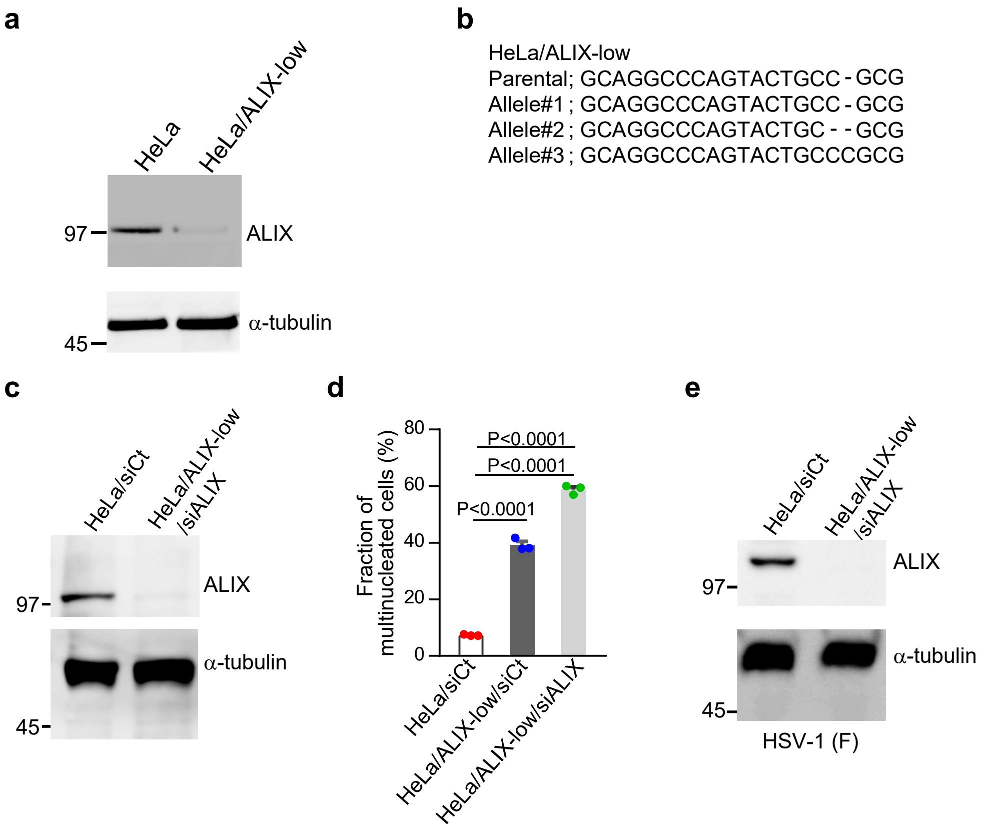

Supplementary Figure 9. Characterization of HeLa/ALIX-low cells. (**a**) Lysates of HeLa and HeLa/ALIX-low cells were analyzed by immunoblotting with anti-ALIX or anti-α-tubulin antibodies. We attempted to generate HeLa cells in which ALIX expression was completely abolished by inactivation of the ALIX gene using the CRISPR/Cas9 system. Although we could not completely abolish ALIX, perhaps because it may be essential in these cultured cells, we were able to obtain cells expressing a significantly reduced level of ALIX protein (ALIX-low cells). The level of ALIX expression in HeLa/ALIX-low cells was 24.7-fold lower than in HeLa cells. Images are representative of 3 independent experiments. (**b**) The targeted ALIX mutation sequences and the parental sequence in HeLa/ALIX-low cells are shown. (**c**) Lysates of HeLa and HeLa/ALIX-low cells were treated with control siRNA (siCt) or ALIX siRNA (siALIX) for 48 h and analyzed by immunoblotting with anti-ALIX and anti-α-tubulin antibodies. The level of ALIX expression in HeLa/ALIX-low cells treated with siRNA to ALIX was 95.2-fold lower than that in HeLa cells treated with control siRNA. Images are representative of 3 independent experiments. (**d**) HeLa or HeLa/ALIX-low cells treated with control siCt or siALIX for 48 h were fixed, stained with both anti-α-tubulin antibodies and Hoechst 33324, and scored for multinucleated cells. Overall, 250-350 cells were analyzed for the presence of more than one nucleus per cell. Data are shown as the mean ± s.e.m. of 3 independent experiments. The indicated P values were obtained using the Tukey's test. (**e**) Lysates of HeLa or HeLa/ALIX-low cells treated with control siCt or siALIX for 48 h respectively were infected with HSV-1 for 22 h, and analyzed by immunoblot with indicated antibodies. Images are representative of 3 independent experiments.

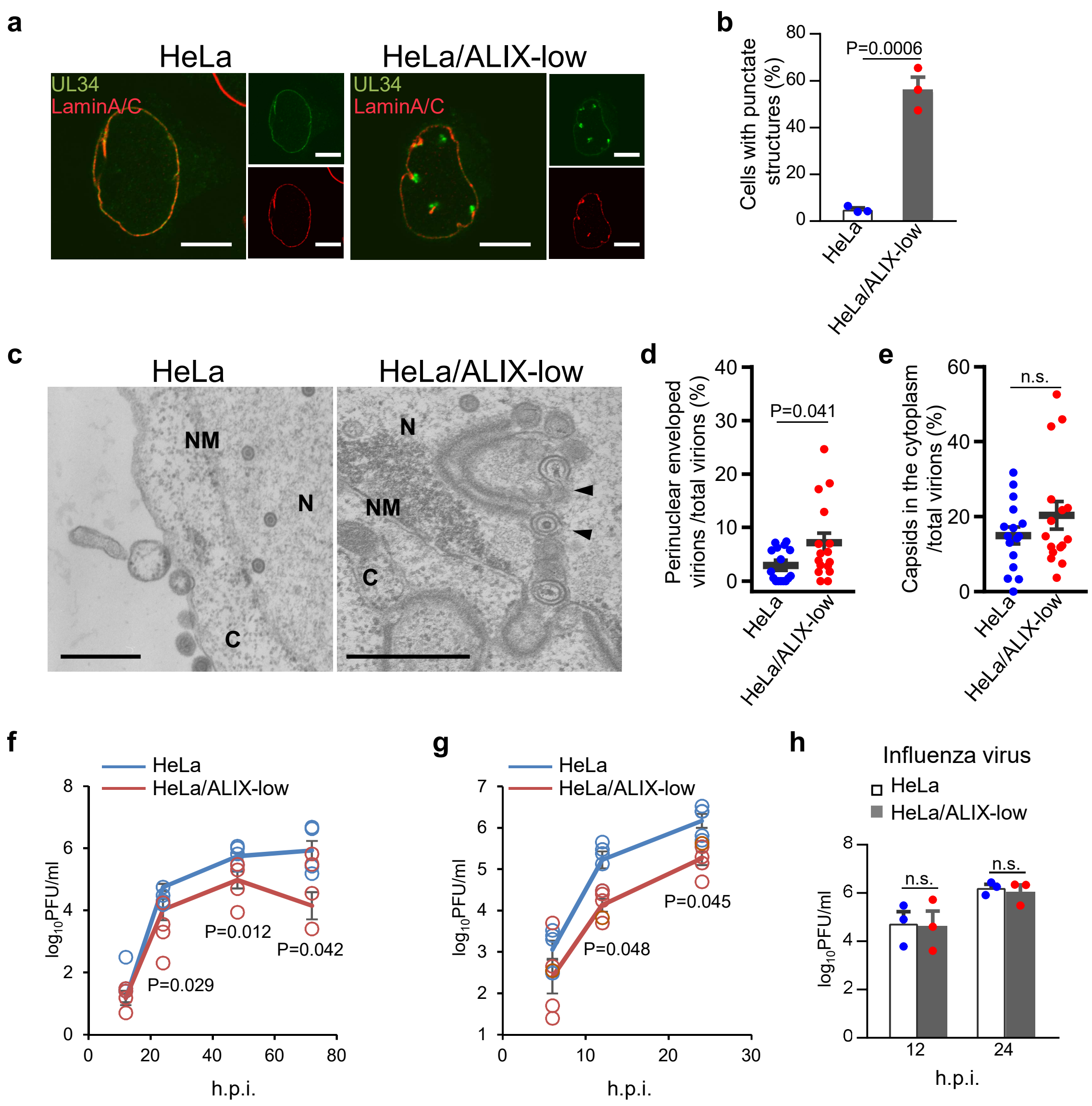

Supplementary Figure 10. Effect of ALIX depletion on the NE budding of HSV-1. (**a**) Confocal microscope images of HeLa and HeLa/ALIX-low cells, infected with HSV-1 for 22 h and stained with anti-lamin A/C and anti-UL34 antibodies. Images are representative of 3 independent experiments. Bars, 20 μm. (**b**) The percent of cells (50-100 cells in each experiment) with aberrant punctate structures along with the nuclear rim was determined. Data are shown as the mean ± s.e.m. of 3 independent experiments. (**c**) Electron microscope images of HeLa and HeLa/ALIX-low cells infected with HSV-1 for 22 h. Arrowheads indicate virions defective in scission steps in the aberrant invagination structures derived from the INM. N, nucleus; C, cytoplasm; NM, nuclear membrane. Bars, 500 nm. Images are representative of 3 independent experiments. The percent of (**d**) perinuclear enveloped virions and (**e**) capsids in the cytoplasm of 16 cells in the experiment in (**c**) was determined. Data are shown as the mean ± s.e.m. and are representative of 3 independent experiments. (**f-h**) HeLa and HeLa/ALIX-low cells were infected with HSV-1 at an MOI of (**f**) 0.05 or (**g**) 10, or with influenza virus at (**h**) an MOI of 0.01, and viral titers were assayed at the indicated hours post-infection (h.p.i.). Data are shown as the mean ± s.e.m. of (**f, g**) 5 or (**h**) 3 independent experiments. The indicated P values were obtained using the unpaired Student's t-test (**b, d-h**). n.s., not significant.

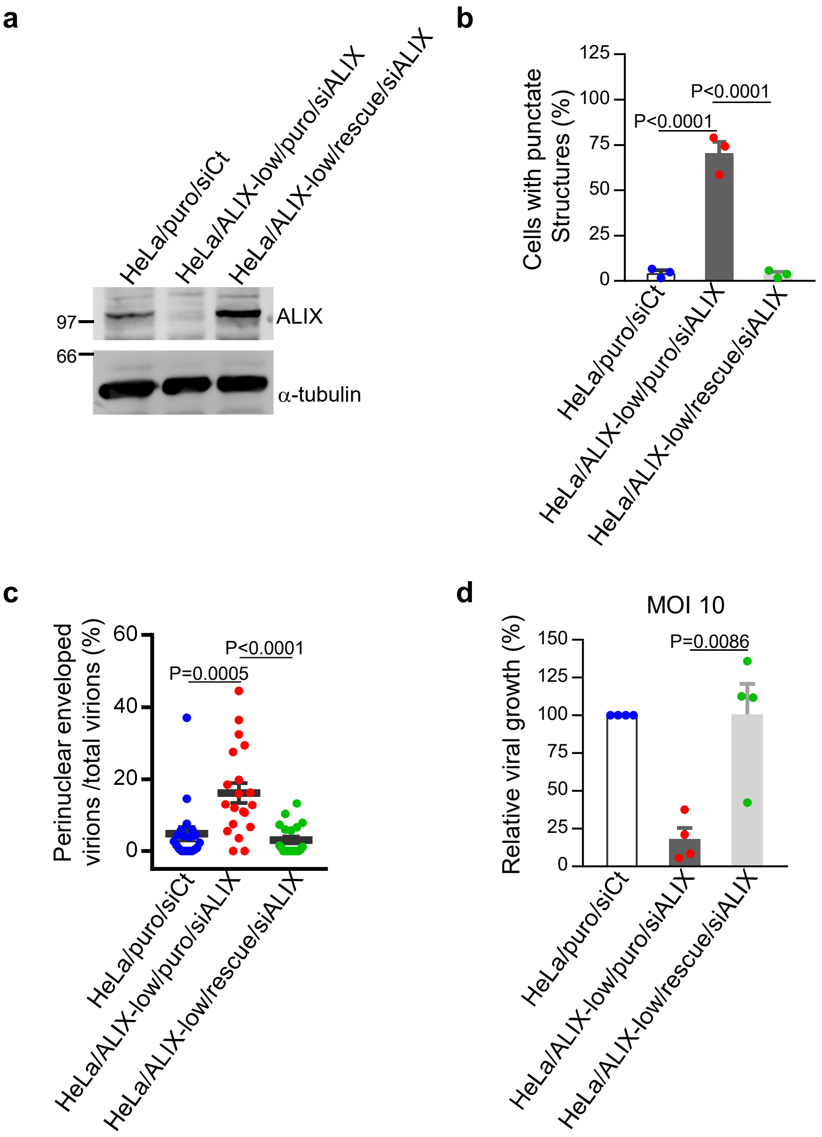

Supplementary Figure 11. Ectopic expression of ALIX rescues the effect of ALIX depletion on NE budding of HSV-1. (**a**) Lysates of HeLa/puro cells treated with control siRNA (siCt), and HeLa/ALIX-low/puro cells and HeLa/ALIX-low/rescue cells treated with siRNAs to ALIX (siALIX) for 48 h were analyzed by immunoblotting with anti-ALIX and anti-α-tubulin antibodies. Images are representative of 3 independent experiments. (**b**) HeLa/puro, HeLa/ALIX-low/puro and HeLa/ALIX-low/rescue cells were treated with siRNA(s) as described for (**a**) and infected with HSV-1 for 22 h. The percent of cells (100-200 cells in each experiment) with aberrant punctate structures along with the nuclear rim was determined. Data are shown as the mean ± s.e.m. of 3 independent experiments. (**c**) HeLa/puro, HeLa/ALX-low/puro and HeLa/ALIX-low/rescue cells were treated with siRNA(s) as described in (**a**) and infected with HSV-1 for 22 h. The percent of perinuclear enveloped virions in 20 cells observed by electron microscopy as described for Fig. 4d was determined. Data are shown as the mean ± s.e.m. and are representative of 3 independent experiments. (**d**) HeLa/puro, HeLa/ALIX-low/puro and HeLa/ALIX-low/rescue cells were treated with siRNA(s) as described in (**a**) and infected with HSV-1 at an MOI of 10 and progeny virus titers were assayed at 24 h post-infection. Data from 4 independent experiments are shown as the mean ± s.e.m. of the relative titer compared to that of HeLa/puro cells treated with siCt. The indicated P values were obtained using the Tukey's test (**b, c**), or the unpaired Student's t-test (**d**).

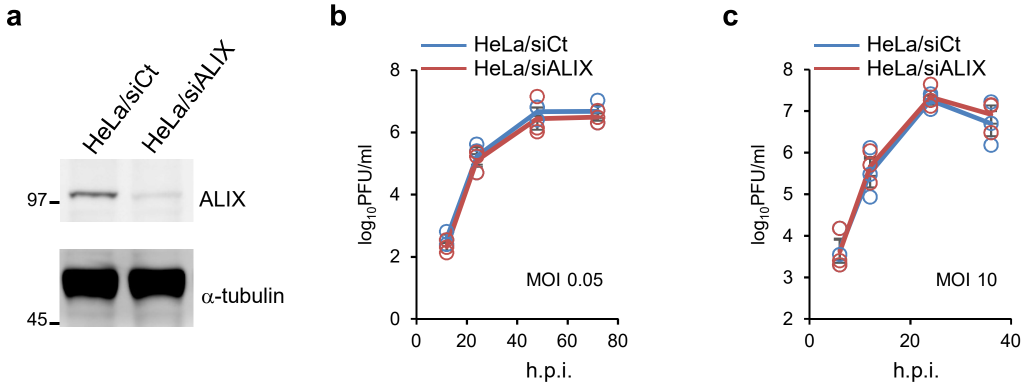

Supplementary Figure 12. Effect of treatment of HeLa cells with siRNA to ALIX on HSV-1 replication. (**a**) Lysates of HeLa cells treated with control siRNA (siCt) or siRNAs to ALIX (siALIX) for 48 h were analyzed by immunoblotting with anti-ALIX and anti-α-tubulin antibodies. Images are representative of 3 independent experiments. (**b,c**) HeLa cells were treated with siRNA(s) as described for (**a**) and infected with HSV-1 at an MOI of (**b**) 0.05 or (**c**) 10. At the indicated hours post-infection (h.p.i.), viral growth was assayed. Data are shown as the mean ± s.e.m. of 3 independent experiments.

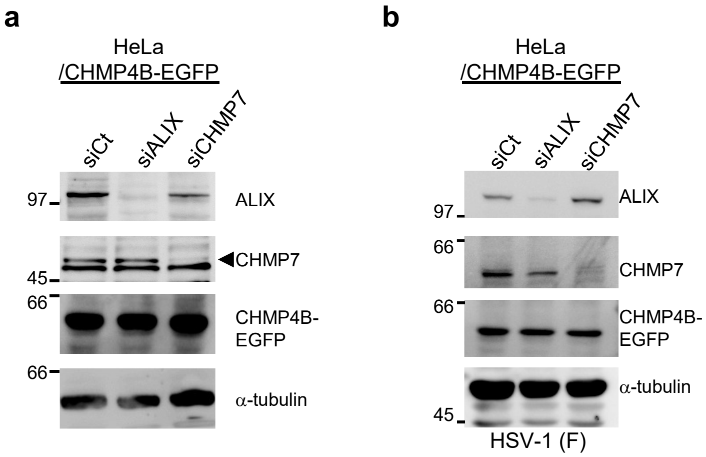

Supplementary Figure 13. Depletion of ALIX or CHMP7 in HeLa/CHMP4B-EGFP cells. Lysates of HeLa/CHMP4B-EGFP cells treated with control siRNA (siCt), siRNA to ALIX (siALIX) or siRNA to CHMP7 (siCHMP7) for 48 h (**a**) and infected with HSV-1 for 22 h (**b**) as in Fig. 5a were analyzed by immunoblotting with anti-ALIX, anti-CHMP7, anti-GFP and anti-α-tubulin antibodies. Images are representative of 3 independent experiments.

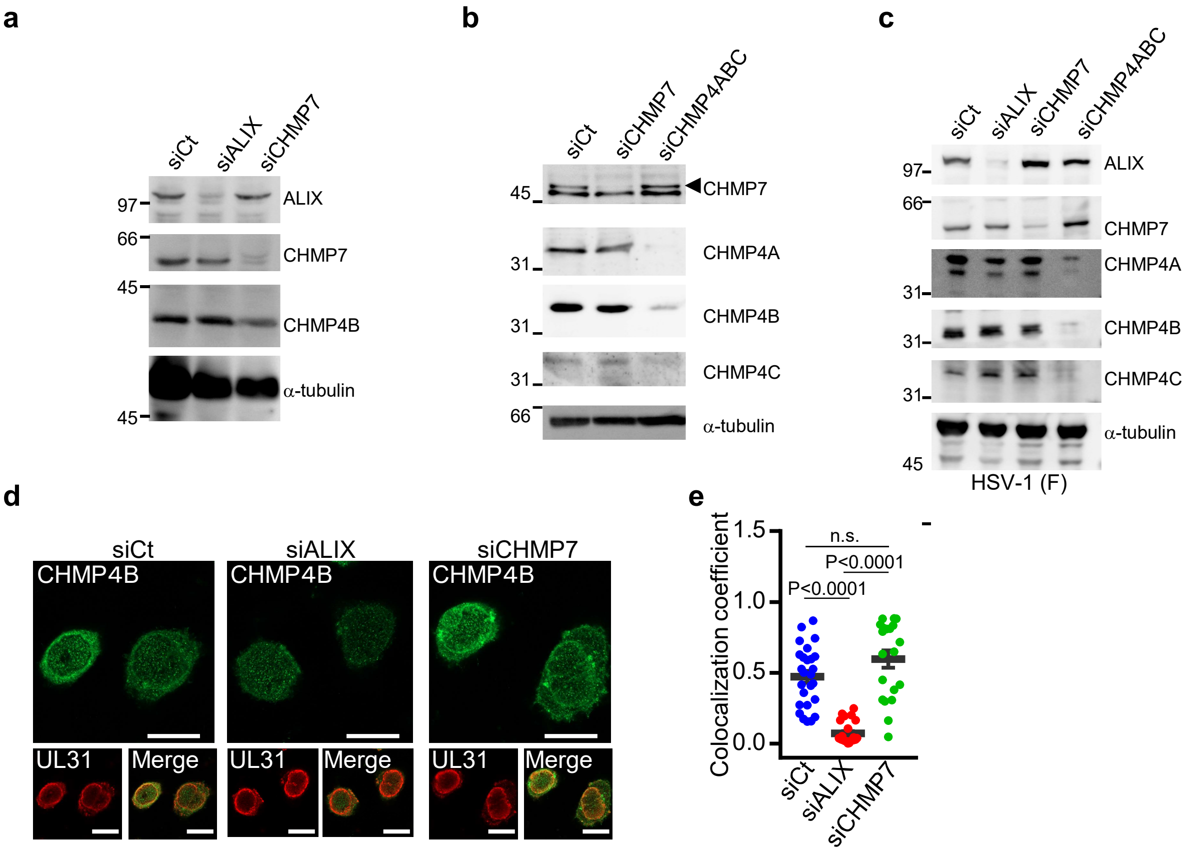

Supplementary Figure 14. Effects of CHMP7 depletion on CHMP4B localization in HSV-1-infected cells. (**a**) Lysates of HeLa cells treated with control siRNA (siCt), siRNA to ALIX (siALIX), or siRNA to CHMP7 (siCHMP7) for 48 h were analyzed by immunoblotting with anti-ALIX, anti-CHMP7, anti-CHMP4B and anti-α-tubulin antibodies. Images are representative of 3 independent experiments. (**b**) Lysates of HeLa cells treated with siCt, siCHMP7, or siRNA to CHMP4A, CHMP4B and CHMP4C (siCHMP4ABC) for 48 h were analyzed by immunoblotting with anti-CHMP7, anti-CHMP4A, anti-CHMP4B, anti-CHMP4C or anti-α-tubulin antibodies. Images are representative of 3 independent experiments. (**c**) Lysates of HeLa cells treated with each of the indicated siRNAs for 48 h and infected with HSV-1 for 22 h were analyzed by immunoblotting with anti-ALIX, anti-CHMP7, anti-CHMP4A, anti-CHMP4B, anti-CHMP4C and anti-α-tubulin antibodies. Images are representative of 3 independent experiments. (**d**) Confocal microscope images of HeLa cells treated with siRNA described in (**a**) for 48 h, infected with HSV-1 for 22 h and stained with anti-CHMP4B and anti-UL31 antibodies. Bars, 20 μm. Images are representative of 3 independent experiments. (**e**) Co-localization between CHMP4B and UL31 was quantified using Mander's co-localization coefficient in the experiment in (**d**). Data are shown as the mean ± s.e.m. (n=26 for siCt, 24 for siALIX or 20 for CHMP7 HSV-1-infected cells) and are representative of 3 independent experiments. The indicated P values were obtained using the Tukey's test. n.s., not significant.

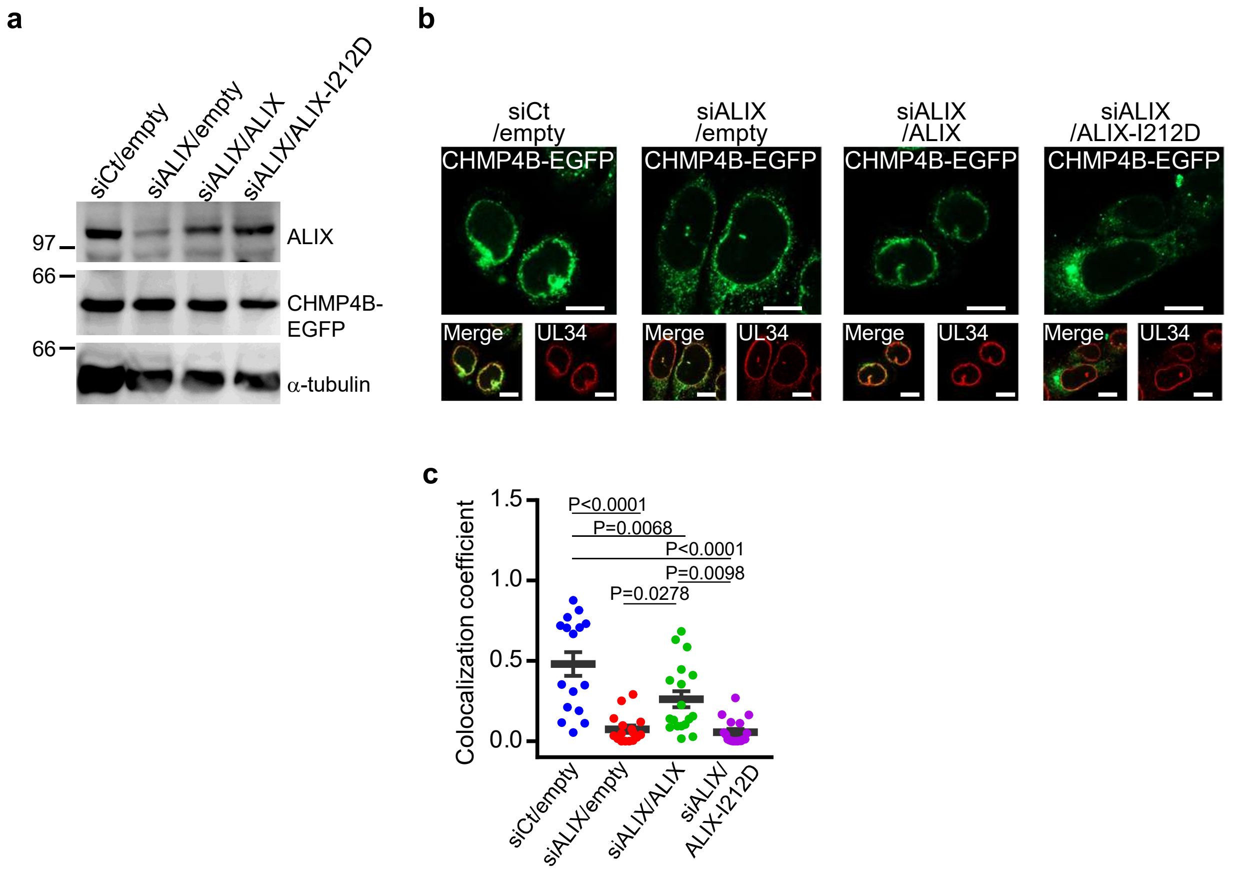

Supplementary Figure 15. Effect of the ectopic expression of wild-type ALIX or ALIX-I212D mutant in ALIX-depleted cells. (**a**) HeLa/CHMP4B-EGFP cells treated with control siRNA (siCt) or siRNA to ALIX (siALIX) for 24 h were transfected with empty vector or each of the indicated ALIX expression vectors for 28 h and analyzed by immunoblotting with anti-ALIX, anti-GFP and anti-α-tubulin antibodies. Images are representative of 3 independent experiments. (**b**) HeLa/CHMP4B-EGFP cells were treated with siCt or siALIX for 24 h, transfected with empty vector or the indicated ALIX expression vector for 6 h, infected with HSV-1 for another 22 h, stained with anti-UL34 antibody and analyzed by confocal microscopy. Images are representative of 3 independent experiments. Bars, 20 μm. (**c**) Co-localization between CHMP4B-EGFP and UL34 was quantified using Mander's co-localization coefficient in the experiment in (**b**). Data are shown as the mean ± s.e.m. (n=16 for cells transfected with control vector or 18 for cells transfected with the ALIX expression vectors) and are representative of 3 independent experiments. The indicated P values were obtained using the Tukey's test.

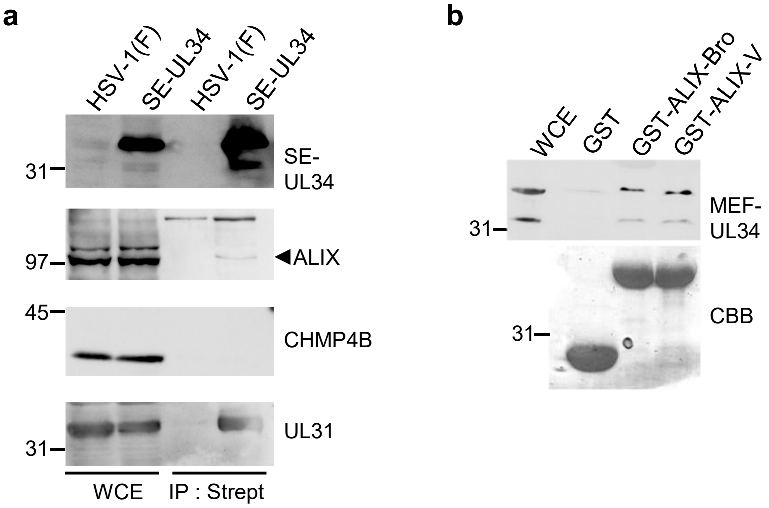

Supplementary Figure 16. Interaction between HSV-1 NEC and ESCRT-III machinery. (**a**) HeLa cells were infected with wild-type HSV-1 or HSV-1 expressing SE-UL34 at an MOI of 0.1 for 48 h, precipitated with StrepTactin Sepharose beads and analyzed by immunoblotting with anti-ALIX, CHMP4B, anti-UL31 and anti-Strep-tag antibodies. Images are representative of 3 independent experiments. WCE; whole cell extract. IP : Strep; precipitates. (**b**) Lysates of Vero cells infected with HSV-1 expressing MEF-UL34 at an MOI of 0.1 for 48 h were reacted with GST, GST-ALIX-Bro or GST-ALIX-V immobilized on Glutathione-Sepharose beads. The beads were then washed extensively and analyzed by immunoblotting with anti-FLAG antibody. Images are representative of 3 independent experiments.

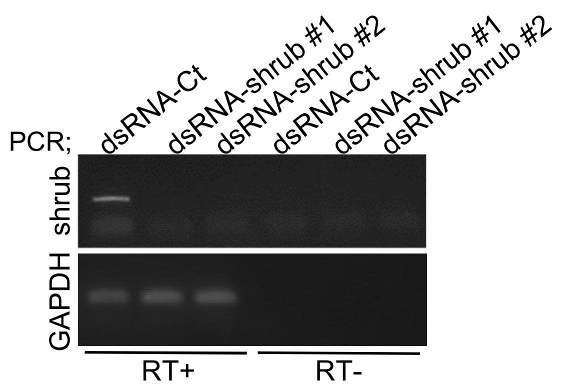

Supplementary Figure 17. Depletion of shrub, a *Drosophila* ortholog of mammalian CHMP4 in S2 cells. Shrub and GAPDH RNA levels in S2 cells treated with control dsRNA (dsRNA-Ct) or dsRNA to shrub (dsRNA-shrub) were assayed by semi-quantitative RT-PCR with (+RT) and without (-RT) reverse transcription. Images are representative of 3 independent experiments.

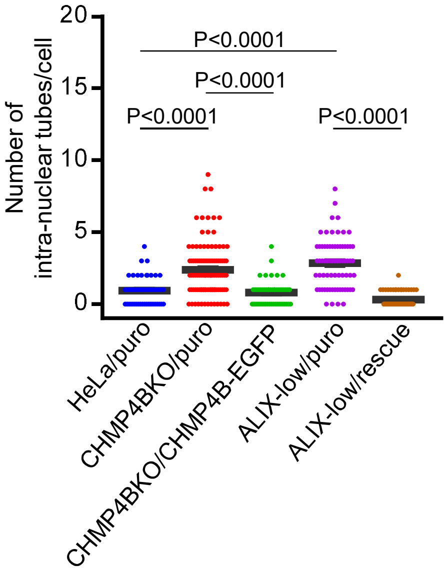

Supplementary Figure 18. Effects of ectopic expression of CHMP4B or ALIX in CHMP4B- or ALIX-depleted cells. The number of trans-nuclear tubes in indicated cell lines was measured (n=50 for HeLa/puro, n=103 for HeLa/CHMP4B KO/puro, n=50 for HeLa/CHMP4B KO/CHMP4B-EGFP, n=72 for HeLa/ALIX-low or n=65 for HeLa/ALIX-low/ALIX). Data are shown as the mean ± s.e.m. and are representative of 3 independent experiments. The indicated P values were obtained using the Tukey's test.

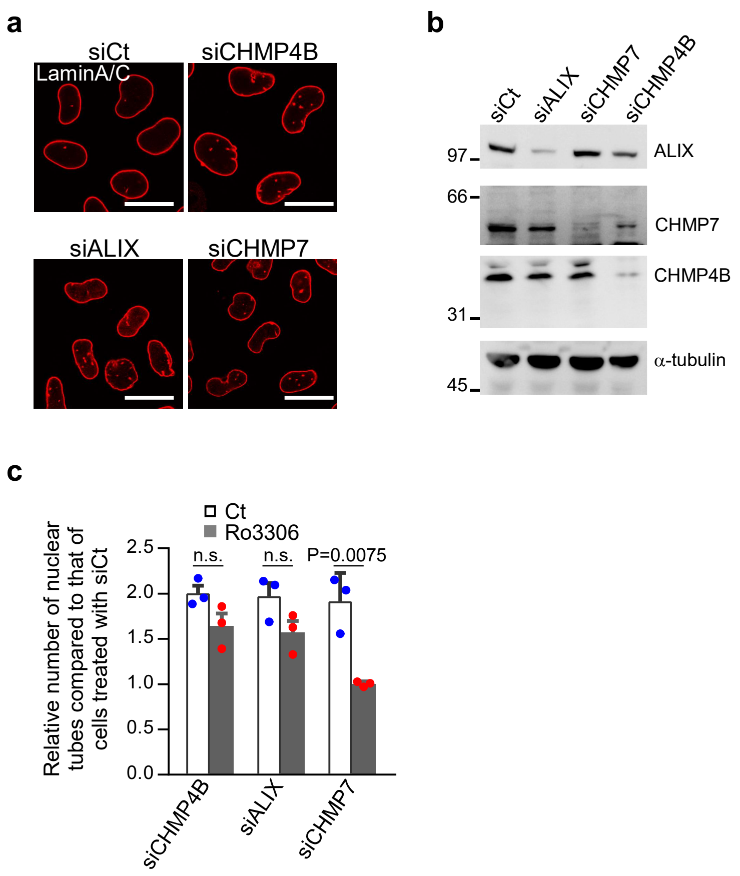

Supplementary Figure 19. Effect of CHMP7 depletion in normal HeLa cells. (**a**) Confocal microscope images of HeLa cells treated with control siRNA (siCt), siRNA to ALIX (siALIX), siRNA to CHMP7 (siCHMP7) or siRNA to CHMP4B (siCHMP4B) for 48 h and stained with anti-Lamin A/C antibody. Bars, 20 µm. Images are representative of 3 independent experiments. (**b**) Lysates of HeLa cells treated with siRNA as described in (**a**) were analyzed by immunoblotting with anti-ALIX, anti-CHMP7, anti-CHMP4B and anti-α-tubulin antibodies. Images are representative of 3 independent experiments. (**c**) Relative number of trans-nuclear tubes of HeLa cells treated with siCHMP4B, siALIX or siCHMP7 with or without cell cycle inhibitor Ro3306 compared to cells treated with siCt. Cells were treated with each of the indicated siRNAs for 24 h and incubated with or without Ro3306 for an additional 24 h. The cells were fixed, stained with anti-Lamin A/C antibody and analyzed by confocal microscopy. The number of trans-nuclear tubes in cells treated with siCHMP4B, siALIX or siCHMP7 was normalized to that of cells treated with siCt in the presence or absence of Ro3306. Data are shown as the mean ± s.e.m. of 3 independent experiments. The indicated P values were obtained using the unpaired Student's t-test. n.s., not significant.

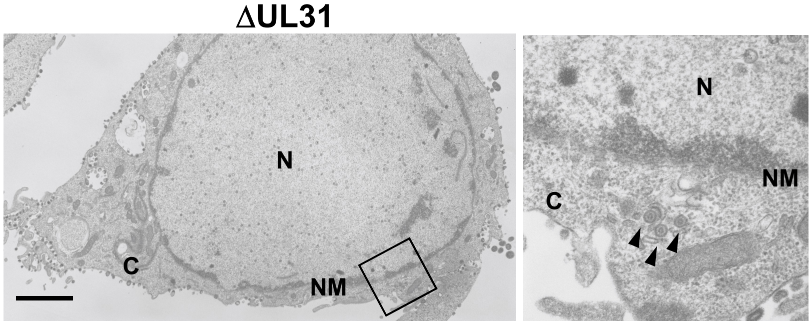

Supplementary Figure 20. Effect of UL31 on the NM integrity in HSV-1-infected rabbit skin cells. Electron microscope images of rabbit skin cells infected with the HSV-1 ΔUL31 at an MOI of 3 for 18 h. The right image is a higher magnification of the boxed area in the left image showing capsids in the cytoplasm (indicated by arrowheads). N, nucleus; C, cytoplasm; NM, nuclear membrane. Bars, 2 µm. Images are representative of 3 independent experiments.

Supplementary Table 1. Effect of KO and KD of CHMP4 proteins on the distribution of virus particles in HSV-1-infected HeLa cells.

Cells	Mean ± standard error of the percent of virus particles in each morphogenetic stage[a]					Total counted (particles/cells)
	Nucleocapsids in nucleus	Virions in perinuclear space	Nucleocapsids in cytoplasm	Enveloped virions in cytoplasm	Extracellular enveloped virions	
HeLa/siCt	31.7 ± 7.5 (576)	2.1 ± 1.1 (39)	8.4 ± 2.1 (154)	3.7 ± 0.9 (68)	53.8 ± 3.2 (975)	1812/14
HeLa/CHMP4B KO/siCHMP4AC	43.3 ± 8.2 (925)	10.7 ± 2.5 (229)	20.1 ± 3.9 (430)	4.8 ± 1.4 (103)	20.8 ± 4.6 (444)	2131/14

[a]Numbers in parentheses are the numbers of virus particles.

Supplementary Table 2. List of primers used in this study.

Name	Sequence
**Construction of pCHMP4B-EGFP-F**	5′-CCGAATTCATGTCGGTGTTCGGGAAGCT-3′
**Construction of pCHMP4B-EGFP-R**	5′-CCGGTACCATGGATCCAGCCCAGTTCT-3′
**Construction of pEGFP-CHMP7-F**	5′-GCAAGCTTCTGGTCCCCGGAGCGGGA-3′
**Construction of pEGFP-CHMP7-R**	5′-GCGGATCCCTACAATGGCTTTAGAGTCG-3′
**Construction of pMxs-ALIX-puro-F**	5′-CCGAATTCACCATGGCGACATTCATCTCGGT-3′
**Construction of pMxs-ALIX-puro-R**	5′-CCGCGGCCGCTTACTGCTGTGGATAGTAAG-3′
**Construction of pcDNA3.1-Flag-ALIX-F**	5′-CCGAATTCACCATGGATTACAAGGATGACGATGACAAGGCGACATTCATC-3′
**Construction of pcDNA3.1-Flag-ALIX-R**	5′-GCGGATCCTTACTGCTGTGGATAGTAAG-3′
**Construction of pGEX-ALIX-Bro-F**	5′-CCGAATTCACCATGGCGACATTCATCTCGGT-3′
**Construction of pGEX-ALIX-Bro-R**	5′-GTCGACCTACATCTTCTCAAACAGATCAG-3′
**Construction of pGEX-ALIX-V-F**	5′-GGGAATTCTCAGTACAGCAGTCTTTGGC-3′
**Construction of pGEX-ALIX-V-R**	5′-GGGTCGACTTATCTTTCTGTCTTCCGTGCAA-3′
**Construction of pX330-CHMP4B-F**	5′-CACCGGGCGGCCCGACCCCCCAGG-3′
**Construction of pX330-CHMP4B-R**	5′-AAACCCTGGGGGGTCGGGCCGCCC-3′
**Construction of pX330-ALIX-F**	5′-CACCGCAGGCCCAGTACTGCCGCG-3′
**Construction of pX330-ALIX-R**	5′-AAACCGCGGCAGTACTGGGCCTGC-3′
**Genotyping of HeLa/CHMP4B-KO cells-F**	5-CCGAATTCATGTCGGTGTTCGGGAAGCT-3′
**Genotyping of HeLa/CHMP4B-KO cells-R**	5′-GGGGCAGTAGCTAGGCAGGT-3′
**Genotyping of HeLa/ALIX-low cells-F**	5′-CCGAATTCACCATGGCGACATTCATCTCGGT-3′
**Genotyping of HeLa/ALIX-low cells-R**	5′-GATGGAAAAATGGGGCGAAAGGG-3′
**Construction of YK540 (SE-UL34)-F**	5′-GAACCCTTTGGTGGGTTTACGCGGGCACGCACGCTCCCATCGCGGGCGCCATGGCTAGCTGGAGCCACCC-3′
**Construction of YK540 (SE-UL34)-R**	5′-TCGAAGGCGTCACCTGGGTGGCCGGTGTAGGGCTTGCCCAGTCCCGCCATACCCTGAAAATACAAATTCT-3′
**Construction of pENTR11-EF-1αp-VPS4-DN-F**	5′-GGGAATTCACCATGGATTACAAGGATGACGATGACAAGACAACGTCAACCCTCCA-3′
**Construction of pENTR11-EF-1αp-VPS4-DN-R**	5′-GGCTCGAGTTAACTCTCTTGCCCAAAGT-3′
**Production of control dsRNA-F**	5′-TAATACGACTCACTATAGGTAAATTGTAAGCGTTAATATTTTG-3′
**Production of control dsRNA-R**	5′-TAATACGACTCACTATAGGAATTCGATATCAAGCTTATCGAT-3′
**Production of shrub#1 dsRNA-F**	5′-TAATACGACTCACTATAGGGATGATCCAGACATGAAG-3′
**Production of shrub#1 dsRNA-R**	5′-TAATACGACTCACTATAGGGTCGATACAAAGCTAAGACTG-3′
**Production of shrub#2 dsRNA-F**	5′-TAATACGACTCACTATAGGGGGAAGATGTTCGGCGGCAAG-3′
**Production of shrub#2 dsRNA-R**	5′-TAATACGACTCACTATAGGGGTTCTCCTGCTCCAGCTCGT-3′
**Semi-quantitative RT-PCR analysis for shrub-F**	5′-ATGAGTTTCTTCGGGAAGAT-3′
**Semi-quantitative RT-PCR analysis for shrub-R**	5′-TTAGTTGGACCAGGATAAAA-3′
**Semi-quantitative RT-PCR analysis for GAPDH-F**	5′-ACTCGACTCACGGTCGTTTC-3′
**Semi-quantitative RT-PCR analysis for GAPDH-R**	5′-GCCGAGATGATGACCTTCTT-3′
